# Salivary proteins, proteases, and disease pathophysiology in oral health and precision diagnostics: a narrative review

**DOI:** 10.3389/fdmed.2026.1931973

**Published:** 2026-08-24

**Authors:** Viorica Rarinca, Cătălina Ionescu, Malina Visternicu, Alin Ciobica, Ionela Vlasie, Anca Trifan, Vasile Burlui, Ancuta Andreea Miler, Andrei Luca, Otilia Novac, Bogdan Novac

**Affiliations:** 1 Doctoral School of Biology, Faculty of Biology, “Alexandru Ioan Cuza” University of Iasi, Iași, Romania; 2 “Ioan Haulica” Institute, Apollonia University, Iasi, Romania; 3 Doctoral School of Geosciences, Faculty of Geography and Geology, “Alexandru Ioan Cuza” University of Iasi, Iași, Romania; 4 Department of Biology, Faculty of Biology, Alexandru Ioan Cuza University of Iasi, Iasi, Romania; 5 “Olga Necrasov” Center, Department of Biomedical Research, Romanian Academy, Iasi, Romania; 6 CENEMED Platform for Interdisciplinary Research, “Grigore T. Popa” University of Medicine and Pharmacy, Iasi, Romania; 7 Department of Dental Medicine, Apollonia University, Iasi, Romania; 8 “Grigore T. Popa” University of Medicine and Pharmacy, Iasi, Romania; 9 Carol Davila University of Medicine and Pharmacy, Bucharest, Romania

**Keywords:** dental biofilm, dental caries, inflammation, oral biochemistry, oral homeostasis, oral microbiota, oxidative stress, periodontal disease

## Abstract

The oral cavity represents a highly complex biochemical ecosystem in which host tissues, saliva, microbial communities, and environmental factors interact dynamically to maintain health or promote disease. This narrative review examines a comprehensive overview of the biochemical processes occurring within the oral cavity and examines their implications for oral health and disease. Key aspects discussed include the biochemical composition and functions of saliva, the metabolic activity of the oral microbiota, biofilm formation, and the molecular mechanisms underlying major oral diseases such as dental caries, periodontal disease, and oral mucosal disorders. Particular emphasis is placed on the role of biochemical homeostasis, microbial dysbiosis, inflammation, and oxidative stress in disease pathogenesis. Although substantial progress has been made in characterizing these mechanisms, current evidence remains largely derived from *in vitro* or reductionist models that do not fully replicate the complexity of the oral environment. In addition, inter-individual variability and host-microbial interactions are still incompletely understood, limiting full translational application. This review also discusses the emerging role of salivary biomarkers as non-invasive diagnostic and prognostic tools, as well as potential therapeutic strategies targeting biochemical pathways. By critically integrating current evidence, this work highlights both advances and remaining gaps in oral biochemistry, emphasizing its relevance for the development of more precise preventive, diagnostic, and personalized approaches in oral healthcare.

## 1 Introduction

The oral cavity represents a complex biological system in which host tissues, microorganisms, and biochemical processes interact continuously within a dynamic and tightly regulated environment (1).

Local defense mechanisms, including salivary antimicrobial factors, immunoglobulins, and epithelial barrier integrity, are essential for maintaining microbial control and tissue integrity (2).

The biochemical environment of the oral cavity is shaped by dynamic interplay between saliva, host tissues, and a highly diverse microbial community (3). Saliva functions not only as a lubricant and digestive fluid, but also as a key regulator of pH, mineral balance, and microbial composition (4). The oral microbiota actively participates in metabolic networks that can support homeostasis or, under dysbiotic conditions, contribute to disease development (5). Together, these interactions sustain oral homeostasis, a fragile equilibrium that is highly sensitive to environmental and host-related perturbations (3).

At the molecular level, oral biochemistry governs essential processes such as mineral ion exchange, protein turnover, redox regulation, and inflammatory signaling (6, 7). These mechanisms ensure the preservation of hard and soft oral tissues under normal conditions and enable adaptive responses to environmental and microbial stressors (5).

Alterations in biochemical regulation shift this balance toward disease via specific macromolecular cascades. In dental caries, this shift is driven by microbial acidogenesis through the Embden-Meyerhof-Parnas pathway, which lowers the local pH below critical threshold (5.5) for hydroxyapatite dissolution (8, 9). In periodontal diseases, dysbiosis triggers host-derived proteolytic cascades, particularly the activation of Matrix Metalloproteinases (MMPs like MMP-8 and MMP-9), alongside a surge in reactive oxygen species, which induce lipid peroxidation and soft tissue degradation (10).

Beyond its local effects, the oral cavity is increasingly recognized for their systemic relevance (3). The oral environment can mirror metabolic, inflammatory, and immunological alterations occurring elsewhere in the body, providing insight into broader health conditions (11). Consequently, the analysis of oral biochemical parameters has emerged as a valuable approach for early disease detection, risk assessment, and monitoring of both oral and systemic disorders (12). This growing body of evidence underscores the significance of oral biochemistry not only in understanding disease mechanisms but also in advancing diagnostic and preventive strategies (13).

The aim of this review is to provide a comprehensive and integrative overview of the biochemical processes occurring within the oral cavity and to examine their implications for oral health and disease. To address current gaps in the literature, this work is structured around four fundamental molecular themes: (1) the thermodynamic and kinetic regulation of salivary buffering and mineral dynamics; (2) the metabolic pathways of the oral biofilm, focusing on acidogenesis and nitrogen catabolism; (3) the specific biochemical mechanisms of disease progression, including matrix metalloproteinase cascades in periodontitis and oxidative stress pathways in mucosal lesions; and (4) the translational utility of advanced salivary omics for molecular diagnostics.

In particular, this review seeks to elucidate the complex and dynamic interactions between host-derived factors and the oral microbiome, emphasizing their role in shaping the biochemical environment of the oral cavity. Key biochemical mechanisms involved in prevalent oral pathologies, including dental caries, periodontal disease, and disorders of the oral mucosa, are critically discussed. Additionally, emerging insights into salivary biomarkers and molecular indicators of disease are highlighted, underscoring their potential utility in diagnosis, risk stratification, and disease monitoring.

Finally, this review aims to explore the translational relevance of oral biochemistry by outlining current and prospective diagnostic, preventive, and therapeutic strategies. By integrating biochemical knowledge with clinical perspectives, this work seeks to contribute to a deeper understanding of oral disease mechanisms and to support the development of targeted approaches for improving oral and overall health.

## 2 Methodology

This study was designed and conducted as a comprehensive narrative review to synthesize current knowledge regarding the biochemical processes governing the oral cavity and their implications for health and disease. A literature search was performed across major electronic databases, including PubMed/MEDLINE, Web of Science (Core Collection), Scopus, and Google Scholar, covering literature published between 2005 and 2026.

The search strategy was structured hierarchically around three main thematic axes: (1) analytical field, (2) biochemical/pathophysiological process, and (3) clinical manifestation. To guarantee exact reproducibility, the specific Boolean search string applied across the database title/abstract/keyword fields was: (“oral biochemistry” OR “salivary proteomics” OR “salivary metabolomics”) AND (“oxidative stress” OR “microbial dysbiosis” OR “mineral homeostasis” OR “proteolysis”) AND (“dental caries” OR “periodontal disease” OR “oral mucosal disease”).

To minimize selection bias, predefined eligibility criteria were strictly enforced. Inclusion criteria were strictly limited to primary, peer-reviewed original research articles, including randomized clinical trials, cohort studies, and case-control designs, as well as *in vitro/in vivo* mechanistic biochemical models. Conversely, exclusion criteria comprised editorial letters, conference abstracts, un-reviewed preprints, secondary literature (such as systematic or narrative reviews, which were utilized solely for background contextualization but excluded from the formal synthesis pool), non-English publications, and studies focusing primarily on systemic diseases without a direct, well-defined, and isolated mechanism.

## 3 The oral cavity as a biochemical ecosystem

The oral cavity functions as a highly dynamic, non-equilibrium biochemical ecosystem driven by complex thermodynamic, fluctuating shear forces, and strict metabolic interdependencies (5). Rather than serving as a static anatomical passage, this microenvironment operates through continuous chemical transitions mediated by the salivary fluid phase, the hard tissue mineral substrate, and the highly organized metabolic activity of the oral biofilm (14).

Saliva represents a central element of this ecosystem, functioning as a dynamic biochemical medium that integrates host-derived molecules with microbial metabolic products and environmental inputs (15). Its continuous secretion and renewal allow saliva to modulate the physicochemical conditions of the oral cavity, including pH, ionic strength, and redox balance.

Specifically, salivary pH regulation is driven by the kinetics of the carbonic anhydrase VI (CA-VI) enzyme, which catalyzes the hydration of carbon dioxide to sustain the bicarbonate buffering system $\left(H_{2}O+CO_{2}⇌\_H_{2}CO_{3}⇌{\mathrm{HCO}}_{3}^{-}+H^{+}\right)$ (16). This chemical equilibrium dictates the thermodynamic saturation ion product (I_P_) of calcium and phosphate relative to the solubility product (K_sp_) of substituted hydroxyapatite, mathematically modulating the driving force for mineral precipitation or dissolution (17). Through these regulatory functions, saliva contributes to the stabilization of the oral environment and supports the maintenance of tissue integrity under constantly changing conditions (18).

Beyond its high-water content and electrolyte composition, saliva contains a wide array of biologically active molecules that perform specialized functions (19). Antimicrobial peptides, immunoglobulins, and other defense-related proteins play a crucial role in controlling microbial growth and preventing pathogen overgrowth. Additionally, growth factors and regulatory peptides present in saliva contribute to epithelial maintenance, wound healing, and tissue regeneration, further emphasizing its role in preserving oral homeostasis (20).

Gingival crevicular fluid further enriches the biochemical landscape of the oral cavity by reflecting the local host response at the tooth-gingiva interface. This fluid contains inflammatory mediators, immune cells, and plasma-derived proteins that are released in response to microbial colonization and mechanical stress (21). At the molecular level, the biochemical signature of GCF during pathological shifts is characterized by an exponential increase in host-derived proteases, particularly active matrix metalloproteinases (MMP-8 and MMP-9), which undergo zymogen activation via cleavage by microbial gingipains. Concurrently, GCF exhibits a depletion of total antioxidant capacity and an elevation of lipid peroxidation products like malondialdehyde, signaling localized oxidative stress in the periodontal niche.

A defining feature of the oral ecosystem is the dynamic and bidirectional interaction between the host, the oral microbiota, and environmental factors, which collectively determine the biochemical characteristics of the oral cavity (22). The oral microbiome consists of a highly diverse, site-specific consortium of microorganisms adapted to distinct ecological niches such as the tooth surface, gingival sulcus, tongue dorsum, and oral mucosa (23). These microbial communities engage in complex metabolic networks that generate a wide range of biochemical products, including organic acids, enzymes, and signaling molecules, thereby exerting a profound influence on the local biochemical environment.

Microbial metabolic activity plays a critical role in modulating parameters such as pH, redox potential, and nutrient availability, which in turn affect both microbial composition and host tissue responses (24). Shifts in microbial metabolism, driven by changes in substrate availability or ecological pressures, can alter biochemical gradients within the oral cavity and promote the selection of pathogenic phenotypes (5). These processes highlight the importance of microbial metabolic balance in maintaining a health-associated oral environment.

Host-derived factors actively regulate microbial colonization and function. Salivary flow rate and composition influence nutrient clearance, buffering capacity, and antimicrobial activity, while epithelial turnover and local immune responses contribute to microbial containment and ecological stability (25).

Oral homeostasis emerges from the finely regulated interplay among host tissues, salivary components, microbial communities, and environmental influences, enabling the oral cavity to preserve structural integrity and functional resilience despite continuous exposure to mechanical, chemical, and microbial stressors (26). This state of equilibrium is sustained through multiple, interdependent biochemical mechanisms that operate in parallel to maintain ecological stability within the oral environment.

Key characteristics of oral homeostasis include controlled microbial growth, balanced metabolic activity, stable pH conditions, effective buffering capacity, and tightly regulated inflammatory responses (5, 27). These processes collectively support enamel integrity, preserve soft tissue architecture, and prevent excessive immune activation. At the molecular level, homeostasis is reinforced by coordinated signaling pathways that regulate antimicrobial defenses, oxidative balance, and tissue remodeling, ensuring an adaptive yet controlled response to external challenges (28).

Disruption of oral homeostasis may occur when biochemical regulation is impaired or when ecological pressures favor microbial dysbiosis (3). Such disturbances can lead to shifts in metabolic outputs, accumulation of harmful byproducts, and sustained inflammatory signaling, thereby promoting tissue damage and disease progression (29). When biochemical regulation fails, the transition from homeostatic resilience to dysbiosis is driven by sustained molecular signaling loops, such as the chronic activation of the Nuclear Factor Kappa B (NF-KB) pathway, which upregulates pro-inflammatory cytokines Interleukin-1 beta (IL-1β) and TNF-α (30). This biomolecular disruption results in a cascade of tissue damage, where excessive reactive oxygen species compromise cellular membranes and disrupt epithelial tight junctions, converting a biochemical imbalance into macroscopic clinical tissue destruction (31).

## 4 Saliva: biochemical composition and functions

Secreted primarily by the major and minor salivary glands, saliva represents a dynamic mixture of water, organic and inorganic components, and biologically active molecules that collectively support oral homeostasis (32). The quantity and quality of salivary secretion are tightly regulated by neural, hormonal, and local factors, allowing saliva to respond rapidly to physiological demands and environmental stimuli (4).

Variations in salivary flow rate and composition exert a significant influence on the physicochemical properties of the oral environment, including pH stability, buffering capacity, and ionic equilibrium. At the molecular level, saliva acts as a supersaturated metastable fluid matrix. Its regulatory capacity is defined by the strict maintenance of an ionic product that exceeds the solubility product of hydroxyapatite, creating a continuous thermodynamic pressure that drives mineral homeostasis at the tooth-fluid interface. Adequate salivary flow facilitates the clearance of food debris and microorganisms, while maintaining optimal conditions for enamel integrity and mucosal protection (33). Conversely, alterations in salivary secretion or biochemical composition may compromise these protective functions, increasing vulnerability to acid-mediated damage, microbial overgrowth, and inflammatory processes (34).

Furthermore, saliva acts as a critical mediator between host tissues and the oral microbiota, shaping microbial adhesion, metabolism, and community structure. By providing nutrients, antimicrobial factors, and signaling molecules, saliva influences microbial behavior and ecological balance within the oral cavity. These interactions underscore the importance of saliva as a determinant of oral health, as disturbances in salivary function are closely associated with an increased risk of dental caries, periodontal disease, and other oral pathologies.

### 4.1 Biochemical composition of saliva

The biochemical composition of saliva reflects its multifunctional nature and underlies its essential role in maintaining oral homeostasis. Although water constitutes the majority of salivary volume, the non-aqueous fraction contains a highly complex mixture of macromolecules and ions that exert significant biological activity (35). The relative concentration of these components varies according to salivary gland origin, flow rate, circadian rhythm, and physiological or pathological conditions, highlighting the dynamic character of saliva (36).

Salivary proteins represent a major functional category and include mucins, proline-rich proteins, statherin, cystatins, and enzymes with structural and regulatory roles (2). Proline-rich proteins and statherin are key constituents of the acquired enamel pellicle and play a critical role in modulating mineral homeostasis by regulating calcium phosphate precipitation and maintaining enamel surface stability (37).

Salivary enzymes such as *α*-amylase, lipase, and various proteases initiate the digestion of dietary macromolecules and influence nutrient availability within the oral cavity (38). Beyond their digestive function, these enzymes can affect microbial adhesion and metabolism, thereby contributing to the ecological balance of the oral microbiome. Additional enzymatic systems, including peroxidases and lysozyme, participate in redox regulation and antimicrobial defense, further emphasizing the biochemical versatility of saliva (39).

Electrolytes constitute another essential component of salivary composition. Calcium and phosphate ions are fundamental for enamel remineralization processes, while bicarbonate and phosphate ions serve as key buffering agents that stabilize oral pH (40). Sodium and potassium ions contribute to osmotic balance and influence salivary secretion dynamics (4). The precise regulation of ionic composition ensures an environment that supports both hard tissue integrity and soft tissue function (41).

Saliva also contains a broad spectrum of antimicrobial peptides and immune-related molecules that form an integral part of the oral defense system (42). Peptides such as defensins, histatins, and cathelicidins exhibit direct antimicrobial activity against bacteria, fungi, and viruses, while secretory immunoglobulin A plays a crucial role in immune exclusion by inhibiting microbial adhesion and colonization. Together, these components bridge innate and adaptive immunity, reinforcing the role of saliva as a key mediator of host defense.

Collectively, the diverse biochemical constituents of saliva enable it to function as an active regulatory fluid rather than a passive secretion. Through coordinated molecular interactions, saliva supports lubrication, digestion, mineral balance, microbial control, and immune surveillance, underscoring its central importance in the maintenance of oral health.

### 4.2 Role of saliva in pH buffering

One of the most important biochemical functions of saliva is its capacity to regulate and buffer pH fluctuations within the oral cavity, thereby preserving a stable chemical environment for oral tissues (18, 43). The buffering action of saliva is primarily mediated by bicarbonate, phosphate, and protein-based buffering systems, which act synergistically to neutralize acids generated by microbial metabolism and dietary intake (43). Among these, the bicarbonate system is considered the most effective, particularly under conditions of increased salivary flow (44).

Salivary buffering capacity is closely associated with flow rate and glandular activity. Stimulated saliva, characterized by elevated bicarbonate concentrations, exhibits enhanced buffering efficiency compared to resting saliva (43). This adaptive response allows the oral cavity to rapidly counteract transient acid challenges following food consumption and microbial fermentation. In addition, salivary proteins and peptides contribute to pH regulation by binding hydrogen ions and modulating the local acid-base balance (34).

By stabilizing oral pH, saliva plays a critical role in protecting enamel and dentin from acid-induced demineralization (45). Maintenance of pH levels above the critical threshold for enamel dissolution favors remineralization processes and limits mineral loss (46). Moreover, a neutral or slightly alkaline oral environment suppresses the proliferation of acidogenic and aciduric microorganisms, thereby influencing microbial composition and metabolic activity within the oral ecosystem (5).

Impairment of salivary buffering capacity, commonly observed in conditions associated with reduced salivary flow or altered salivary composition, can lead to prolonged acidic conditions in the oral cavity (47). Such environments promote enamel erosion, enhance microbial acid production, and increase susceptibility to dental caries. Consequently, the regulation of oral pH through salivary buffering represents a fundamental protective mechanism and a key determinant of oral health.

### 4.3 Protective, digestive, and remineralization functions

Saliva performs a wide range of protective functions that are essential for the preservation of both hard and soft oral tissues (48). Through its lubricating properties and the formation of the acquired salivary pellicle, saliva minimizes mechanical abrasion during mastication and speech while reinforcing the integrity of the mucosal barrier (49). This pellicle acts as a selective interface between the tooth surface and the oral environment, modulating mineral exchange and limiting direct exposure to erosive and microbial challenges (50). In parallel, salivary antimicrobial components and immune mediators inhibit pathogen adhesion, aggregation, and colonization, thereby reducing the risk of infection and limiting excessive inflammatory responses that can lead to tissue damage (34).

In addition to its protective role, saliva plays a critical part in the initiation of the digestive process (38). Salivary enzymes such as *α*-amylase and lipase initiate the breakdown of carbohydrates and lipids, contributing to early digestive processes and influencing nutrient availability in the oral cavity (38). Saliva also supports gustatory function by dissolving tastants and transporting them to taste receptors, thereby linking sensory perception with digestive physiology (45, 51).

Saliva further contributes to the maintenance of dental hard tissues through its central role in enamel remineralization (45). By maintaining a state of supersaturation with respect to calcium phosphate minerals, saliva promotes the deposition of mineral ions onto demineralized enamel surfaces (37, 52). Specific salivary proteins, including statherin and proline-rich proteins, regulate mineral precipitation and crystal growth, preventing uncontrolled calcification while supporting targeted remineralization of early lesions (37). This dynamic balance between demineralization and remineralization is essential for preserving enamel integrity over time.

Collectively, the protective, digestive, and remineralization functions of saliva underscore its role as a key determinant of oral health (33). Disruption of these functions, whether through quantitative or qualitative alterations in salivary secretion, can compromise tissue resilience and increase susceptibility to oral diseases (22). Thus, saliva represents a central biological factor in maintaining oral function and preventing pathological processes.

### 4.4 Critical perspectives on biofilm dynamics

Although the biochemical stages of biofilm formation have been extensively characterized, major challenges persist in understanding its dynamic behavior within the oral ecosystem. Most current models rely predominantly on static *in vitro* systems that inadequately reproduce the complex hydrodynamic conditions and continuous nutrient fluctuations of the oral cavity (53). Importantly, the transition from a commensal to a pathogenic biofilm should not be interpreted solely as a shift in microbial composition, but rather as a coordinated metabolic adaptation driven by environmental stress, ecological competition, and sustained pH alterations (54). In addition, considerable inter individual variability in acquired pellicle composition remains insufficiently explored, despite its critical role in mediating the initial biochemical interactions between host surfaces and pioneer colonizers. These limitations highlight the need for a paradigm shift in therapeutic strategies, moving beyond indiscriminate microbial reduction toward targeted modulation of biofilm associated metabolic pathways. Such a perspective may ultimately support the development of precision based preventive and therapeutic approaches, including next generation probiotics and highly selective antimicrobial interventions.

## 5 Oral microbiota and biofilm formation

The oral microbiota constitutes a highly diverse and dynamic microbial community that plays a fundamental role in shaping the biochemical environment of the oral cavity. This community is composed of hundreds of microbial species that coexist within structured ecological networks, engaging in complex metabolic and signaling interactions (55).

Under physiological conditions, the oral microbiota exists in a balanced state characterized by controlled microbial growth and cooperative metabolic activity. Commensal microorganisms participate in colonization resistance by occupying ecological niches and limiting the expansion of opportunistic pathogens (3, 56). Through their metabolic functions, these microbes contribute to nutrient cycling, pH modulation, and redox balance, thereby supporting a stable oral ecosystem compatible with health (57).

However, alterations in microbial composition, spatial organization, or metabolic activity can disrupt this balance and promote disease development. Ecological shifts driven by changes in substrate availability, host defenses, or environmental factors may favor the emergence of pathogenic microbial communities with enhanced acidogenic, proteolytic, or inflammatory potential (22). Such dysbiotic states are associated with the accumulation of harmful biochemical byproducts and sustained inflammatory signaling, which can compromise enamel integrity and soft tissue health (28). These observations underscore the critical role of microbial-biochemical interactions in determining whether the oral microbiota functions as a protective or pathogenic component of the oral ecosystem.

### 5.1 Composition of the oral microbiota

The oral microbiota is composed of a complex and heterogeneous consortium of microorganisms, including bacteria, fungi, viruses, and archaea, that colonize distinct ecological niches within the oral cavity (58). These niches, such as the tooth surface, gingival sulcus, tongue dorsum, and oral mucosa, are characterized by unique physicochemical parameters, including oxygen availability, nutrient gradients, pH, and shear forces. Such site-specific conditions exert selective pressures that shape the composition and functional specialization of local microbial communities (59).

Bacterial species dominate the oral microbiome and represent the most extensively studied component. In healthy individuals, the oral bacterial community is typically enriched with genera such as *Streptococcus*, *Actinomyces*, *Veillonella*, *Fusobacterium*, and *Porphyromonas*, which coexist in a balanced and cooperative manner (3, 60). Early colonizers, particularly streptococci and actinomycetes, play a crucial role in establishing microbial communities by adhering to oral surfaces and creating conditions that facilitate the attachment of subsequent microbial species (61). Later colonizers contribute to increased community complexity and functional diversity.

In addition to bacteria, the oral microbiota includes fungal species, most notably *Candida*, which may exist as commensals or opportunistic pathogens depending on host and environmental conditions (62). Viruses, including bacteriophages, influence microbial population dynamics by modulating bacterial abundance and gene transfer, while archaea, though less abundant, have been detected in specific niches such as periodontal pockets and may contribute to anaerobic metabolic processes (63).

Microbial composition varies not only between anatomical sites but also among individuals and across the lifespan, reflecting the influence of host genetics, immune status, dietary habits, oral hygiene practices, and environmental exposures (57). In a healthy oral ecosystem, commensal microorganisms contribute to colonization resistance by competing with potential pathogens for nutrients and adhesion sites and by participating in metabolic interactions that support ecological stability (5). These cooperative and competitive relationships underscore the complexity of the oral microbiota and its central role in maintaining oral health.

### 5.2 Bacterial metabolism and biochemical products

Bacterial metabolic activity represents a central determinant of the biochemical conditions within the oral cavity and plays a pivotal role in shaping both microbial ecology and host tissue responses (5, 64). Oral microorganisms metabolize a wide spectrum of substrates, including dietary carbohydrates, proteins, and host-derived compounds such as glycoproteins and peptides present in saliva and gingival crevicular fluid (22). The nature and availability of these substrates strongly influence microbial metabolic pathways and the resulting biochemical outputs.

Carbohydrate metabolism by oral bacteria is particularly relevant to oral health, as fermentation processes generate organic acids, most notably lactic acid, that can significantly reduce local pH (65). Sustained acid production promotes enamel demineralization and selects for acid-tolerant microbial species, contributing to ecological shifts associated with dental caries (8). In contrast, the metabolism of nitrogen-containing compounds, including urea and amino acids, can lead to the production of alkaline byproducts such as ammonia, which partially neutralize acidic conditions and contribute to pH homeostasis under certain circumstances (66).

Beyond acid-base modulation, bacterial metabolism produces a broad array of bioactive molecules that influence host-microbe interactions (67). These include enzymes involved in proteolysis and glycan degradation, short-chain fatty acids, and various metabolic intermediates that act as signaling molecules (67, 68). Such products can modulate epithelial barrier integrity, alter immune cell activation, and influence inflammatory signaling pathways. While some metabolic byproducts support tissue health and microbial balance, others may promote inflammation and tissue breakdown when produced in excess or under dysbiotic conditions (69).

The overall impact of bacterial metabolism on oral health is therefore determined by the balance between protective and pathogenic biochemical activities. A stable and health-associated metabolic profile supports ecological equilibrium and tissue integrity, whereas shifts toward harmful metabolic outputs can drive disease initiation and progression. Understanding these metabolic processes is essential for elucidating the biochemical mechanisms underlying oral diseases and for identifying potential targets for therapeutic intervention.

### 5.3 Dental biofilm formation

A hallmark of oral microbial organization is the formation of structured biofilms, commonly referred to as dental plaque, which represent highly organized and functionally integrated microbial communities (70). Biofilm formation is a tightly regulated, multistep process that begins with the adsorption of salivary proteins and glycoproteins onto the tooth surface, resulting in the formation of the acquired enamel pellicle (71). This proteinaceous layer provides specific binding sites for early colonizing bacteria, which adhere through highly selective adhesin-receptor interactions.

Early colonizers, predominantly facultative anaerobic bacteria, establish the initial microbial layer and modify the local environment in ways that promote further microbial attachment (72). These microorganisms produce extracellular molecules and alter oxygen availability, facilitating the recruitment of secondary colonizers and increasing microbial diversity. As the biofilm develops, microbial succession leads to the establishment of complex, multispecies communities with spatial and functional organization (73).

As the biofilm matures, microbial cells become embedded in an extracellular polymeric substance matrix composed primarily of polysaccharides, proteins, lipids, and extracellular DNA (74). This matrix serves multiple functions, including structural cohesion, retention of nutrients, and protection against mechanical disruption, antimicrobial agents, and host immune defenses (75). The biofilm architecture creates localized microenvironments characterized by gradients in pH, oxygen tension, and nutrient concentration, which influence microbial metabolism and gene expression (76).

Within the biofilm, microorganisms engage in metabolic cooperation and intercellular communication through mechanisms such as quorum sensing. These interactions enable coordinated responses to environmental changes and contribute to the persistence and adaptability of the microbial community (76, 77). However, under conditions that favor dysbiosis, biofilm-associated metabolic activity can lead to the accumulation of harmful biochemical byproducts, sustained inflammation, and tissue damage (78).

The accumulation and maturation of dental biofilm can shift the oral ecosystem toward pathogenic states, particularly when ecological pressures favor acidogenic or proteolytic species (79). Biofilm-associated microorganisms exhibit increased resistance to antimicrobial agents and host immune responses, contributing to the chronic nature of biofilm-related oral infections (80). Consequently, understanding the biochemical, structural, and ecological dynamics of dental biofilm formation is essential for elucidating the mechanisms underlying dental caries, periodontal disease, and other biofilm-associated oral pathologies, as well as for developing effective preventive and therapeutic strategies (80, 81).

## 6 Biochemical mechanisms in oral diseases

### 6.1 Dental caries

Dental caries is a multifactorial, biofilm-mediated disease driven by complex biochemical interactions between dietary substrates, oral microorganisms, and host factors (8). At its core, caries development reflects a sustained imbalance between demineralization and remineralization processes at the tooth surface, largely governed by microbial metabolism and the biochemical properties of the oral environment (82).

### 6.2 Carbohydrate metabolism in dental caries

The metabolism of dietary carbohydrates by oral bacteria represents a central biochemical mechanism in the initiation and progression of dental caries. Fermentable carbohydrates, particularly sucrose, glucose, and fructose, serve as primary energy sources for cariogenic microorganisms (83). Through glycolytic pathways, these substrates are rapidly metabolized, resulting in the production of organic acids. Sucrose holds a unique role in cariogenesis, as it not only fuels acid production but also serves as a substrate for the synthesis of extracellular polysaccharides that enhance bacterial adhesion and biofilm structural integrity (84).

Frequent exposure to fermentable carbohydrates promotes prolonged acidogenic conditions within dental biofilms, selecting for aciduric bacterial species capable of surviving and functioning at low pH levels (8). This ecological shift reinforces a dysbiotic biofilm composition and sustains biochemical conditions conducive to tooth mineral loss.

From a critical biochemical standpoint, this ecological shift is heavily driven by the regulation of specific bacterial enzymes, such as glucosyltransferases (GTFs) produced by *Streptococcus mutans*, which synthesize glucans from sucrose (85). Clinically, this process alters the physical properties of the dental plaque, turning it into a dense, hydrophobic matrix that limits the buffering capacity of saliva (135). Consequently, the critical pH threshold of 5.5 is crossed for extended periods, shifting the thermodynamic balance of hydroxyapatite toward dissolution (86). Understanding these precise enzymatic and metabolic pathways highlights why targeting bacterial adhesion and plaque acidogenicity, rather than merely reducing general bacterial load, remains a pivotal strategy in modern preventive dentistry (8).

### 6.3 Acid production and enamel demineralization

Acid production resulting from bacterial carbohydrate metabolism leads to a localized drop in pH at the tooth-biofilm interface (87). When pH levels fall below the critical threshold for enamel stability, mineral ions such as calcium and phosphate are released from the enamel surface, initiating demineralization (88). Repeated or prolonged acid challenges impair the natural remineralization capacity of saliva, tipping the balance toward net mineral loss (89).

The extent of enamel demineralization is influenced by the intensity and duration of acidic episodes, as well as by salivary buffering capacity and mineral availability (45). In early stages, demineralization may be reversible; however, sustained biochemical disruption and biofilm maturation can result in irreversible structural damage and cavitation (90). Thus, the biochemical processes of carbohydrate metabolism and acid production are fundamental drivers of caries pathogenesis, linking dietary habits, microbial activity, and host defense mechanisms.

Crucially, this demineralization dynamics is governed by the saturation state of saliva with respect to hydroxyapatite [Ca_10_(PO_4_)_6_(OH)_2_] and fluorapatite [Ca_10_(PO_4_)_6_F_2_] (91). When the pH drops below the critical threshold of approximately 5.5 for enamel, saliva becomes undersaturated with respect to hydroxyapatite, driving the thermodynamic dissolution of calcium and phosphate ions into the biofilm fluid (136). From a clinical and therapeutic perspective, this biochemical vulnerability underscores the critical role of topical fluorides and biomimetic remineralizing agents (such as nano-hydroxyapatite or casein phosphopeptide-amorphous calcium phosphate, CPP-ACP) (92). These agents artificially maintain a state of supersaturation at the tooth-biofilm interface even under moderate acidic challenges, effectively shifting the equilibrium back toward remineralization before cavitation occurs (93). Evaluating these dynamic ion-exchange gradients represents a cornerstone for developing modern, non-invasive therapies in personalized cariology (94).

### 6.4 Periodontal disease

Periodontal disease encompasses a group of chronic inflammatory conditions affecting the supporting tissues of the teeth, including the gingiva, periodontal ligament, and alveolar bone (95). Unlike dental caries, which is primarily driven by acid-mediated mineral loss, periodontal disease is characterized by a dysregulated host inflammatory response to a dysbiotic subgingival biofilm (96). Biochemical mechanisms play a central role in mediating tissue destruction and disease progression.

### 6.5 Inflammatory processes in periodontal disease

Inflammation represents a key biochemical hallmark of periodontal disease and arises from sustained microbial challenge within the gingival sulcus (97). Periodontal pathogens and their virulence factors, such as lipopolysaccharides and other microbial-associated molecular patterns, activate host immune cells and epithelial receptors, triggering the release of pro-inflammatory cytokines and chemokines (98). These mediators promote leukocyte recruitment, vascular permeability, and the amplification of inflammatory signaling cascades.

While inflammation initially serves a protective function, chronic and excessive inflammatory responses result in collateral tissue damage (99). The persistent production of inflammatory mediators alters local biochemical conditions, increases oxidative stress, and disrupts the balance between tissue breakdown and repair (100). This sustained inflammatory milieu contributes to progressive periodontal attachment loss and bone resorption.

### 6.6 Extracellular matrix degradation

The destruction of periodontal tissues is closely linked to biochemical degradation of the extracellular matrix, which provides structural support to the gingiva and periodontal ligament. During periodontal disease, host-derived enzymes and inflammatory mediators promote the breakdown of collagen fibers, proteoglycans, and other matrix components (101). This degradation weakens tissue integrity and facilitates further microbial invasion.

Matrix degradation is further exacerbated by imbalances between proteolytic enzymes and their endogenous inhibitors (102). Under pathological conditions, regulatory mechanisms that normally limit tissue breakdown become impaired, allowing excessive extracellular matrix destruction and irreversible periodontal damage.

### 6.7 Role of proteolytic enzymes

Proteolytic enzymes play a critical role in periodontal tissue breakdown and disease progression (103). These enzymes originate from both host immune cells and periodontal pathogens. Host-derived matrix metalloproteinases are particularly important mediators of collagen degradation and are upregulated in response to inflammatory stimuli. When their activity exceeds inhibitory control, these enzymes contribute significantly to connective tissue destruction (101).

Microbial proteases further amplify tissue damage by degrading host proteins, disrupting immune defenses, and generating bioactive fragments that enhance inflammation (104). The combined action of host and bacterial proteolytic enzymes establishes a self-perpetuating cycle of inflammation and tissue degradation. Understanding the biochemical regulation of proteolytic activity is therefore essential for elucidating the pathogenesis of periodontal disease and identifying potential therapeutic targets (97). To provide a comprehensive biochemical mapping of these destructive cascades, the prominent host-derived and microbial proteolytic enzymes driving periodontal tissue breakdown are systematically integrated in Table 1. Host-derived enzymes such as MMP-8, released primarily by neutrophils and fibroblasts, directly cleave the triple helix of interstitial collagen and serve as a primary biomarker for active periodontal destruction (105). Microbial proteases produced by key periopathogens such as *Porphyromonas gingivalis* (gingipaines Rgp and Kgp) disrupt host immune defenses by degrading antibodies and complement components while activating host pro-MMPs to drive a destructive cascade (106). Lysosomal proteases from neutrophils, including neutrophil elastase (NE) and cathepsin G, destroy non-collagenous matrix components during acute inflammatory spikes and further amplify tissue breakdown (107). Critical evaluation of these enzymatic pathways highlights a synergistic, dual-source mechanism of destruction (108). This enzymatic interplay extends beyond simple tissue degradation; microbial proteases cleave host immunoglobulins and complement components, thereby neutralizing immune surveillance, while simultaneously processing and activating host pro-MMPs (109). Consequently, this bidirectional feedback loop transitions the periodontal microenvironment from localized inflammation to self-perpetuating, irreversible tissue and alveolar bone resorption, emphasizing the potential of specific proteolytic profiles as targeted diagnostic and therapeutic biomarkers.

**Table 1:** Host-derived and microbial proteolytic enzymes in periodontal tissue destruction.

Enzyme class and source	Specific enzymes	Primary biochemical substrates	Pathogenic role and tissue impact	References
Host-Derived *(Immune cells, mainly PMNs and fibroblasts)*	MMP-8 (Collagenase-1) MMP-9 (Gelatinase-B)	Type I and Type III collagens (the structural backbone of the periodontium). Gelatin, collagen fragments, and elastin.	Directly cleaves the triple helix of interstitial collagen. Drives the irreversible degradation of the ECM and alveolar bone loss. Used as a primary biomarker for active periodontal destruction.	(105)
Microbial/Bacterial *(Key periopathogens, e*.*g*.*, Porphyromonas gingivalis)*	Gingipains: Rgp (Arginine-specific) Kgp (Lysine-specific)	Host structural proteins (collagen, fibronectin), immunoglobulins (IgG, IgA), and complement factors.	Disrupts host immune defenses by degrading antibodies and complement components. Activates host pro-MMPs (creating a destructive cascade). Increases vascular permeability, leading to increased GCF flow to feed asaccharolytic bacteria.	(106)
Host-Derived *(Lysosomal proteases from neutrophils)*	NE Cathepsin G	Elastin, proteoglycans, and fibronectin.	Destroys non-collagenous matrix components during acute inflammatory spikes. Amplifies inflammation by processing and activating specific pro-inflammatory cytokines (like IL-1β).	(107)

ECM, extracellular matrix; IL-1β; GCF, gingival crevicular fluid; IgA, immunoglobulin A; IgG, immunoglobulin G; IL-1β, Interleukin 1 beta; Kgp, lysine specific gingipain; MMP-8, matrix metalloproteinase-8; MMP-9, matrix metalloproteinase-9; NE, neutrophil elastase; PMNs, polymorphonuclear leukocytes; Rgp, arginine-specific gingipain.

### 6.8 Oral mucosal diseases

Oral mucosal diseases comprise a heterogeneous group of conditions affecting the epithelial lining and underlying connective tissues of the oral cavity (110). These disorders include inflammatory, infectious, autoimmune, and potentially malignant lesions, many of which are characterized by profound biochemical alterations at the cellular and molecular levels (111). Changes in epithelial metabolism, immune signaling, and redox balance play a central role in disease onset and progression.

### 6.9 Biochemical alterations of the oral mucosa

The oral mucosa is a metabolically active tissue that relies on tightly regulated biochemical processes to maintain barrier function and tissue integrity. In mucosal diseases, disruptions in cellular signaling pathways, protein expression, and lipid metabolism alter epithelial differentiation and turnover. Abnormal expression of cytokines, growth factors, and adhesion molecules can compromise epithelial cohesion and increase tissue permeability (112).

Alterations in salivary composition and microbial metabolites further influence mucosal biochemistry by modulating local pH, enzymatic activity, and immune responses. These changes can promote epithelial injury and impair wound healing, contributing to the persistence and recurrence of mucosal lesions. Biochemical dysregulation at the mucosal surface therefore represents both a cause and a consequence of disease-related tissue damage.

### 6.10 Oxidative stress and chronic inflammation

Oxidative stress is a key biochemical mechanism implicated in the pathogenesis of many oral mucosal diseases. Excessive production of reactive oxygen and nitrogen species, often driven by chronic inflammation and immune cell activation, overwhelms local antioxidant defense systems (113). This imbalance leads to oxidative damage of cellular components, including lipids, proteins, and nucleic acids, resulting in impaired cell function and increased susceptibility to apoptosis or malignant transformation.

Chronic inflammation sustains oxidative stress through continuous cytokine release and immune cell infiltration, creating a self-reinforcing pathological environment. Prolonged exposure to oxidative and inflammatory mediators can induce genetic and epigenetic alterations in epithelial cells, contributing to disease progression and, in some cases, carcinogenesis. Understanding the biochemical interplay between oxidative stress and inflammation is therefore essential for elucidating the mechanisms underlying oral mucosal diseases and for identifying biomarkers and therapeutic strategies aimed at restoring mucosal homeostasis.

## 7 Salivary biomarkers in oral diseases

Saliva has emerged as a promising diagnostic fluid due to its rich biochemical composition and its close association with physiological and pathological processes occurring within the oral cavity. As a complex mixture of salivary gland secretions, gingival crevicular fluid, epithelial cells, microorganisms, and locally derived inflammatory mediators, saliva provides a comprehensive snapshot of biological activity at the level of oral tissues (114). This multifaceted origin enables saliva to reflect pathological changes arising from dental, periodontal, and oral mucosal structures, making it particularly relevant for the assessment of oral diseases.

Advances in analytical technologies, including proteomics, metabolomics, genomics, and immunoassay-based platforms, have significantly expanded the scope of salivary diagnostics in oral medicine. These approaches allow for the sensitive and precise identification and quantification of a wide range of salivary biomarkers, even at low concentrations. Consequently, saliva is increasingly recognized as a valuable medium for the early detection of oral diseases, assessment of disease activity, and monitoring of therapeutic outcomes. The growing interest in saliva-based diagnostics highlights its potential to complement, and in some contexts replace, more invasive diagnostic methods used in oral healthcare.

### 7.1 Biochemical salivary biomarkers

**Figure 1:**
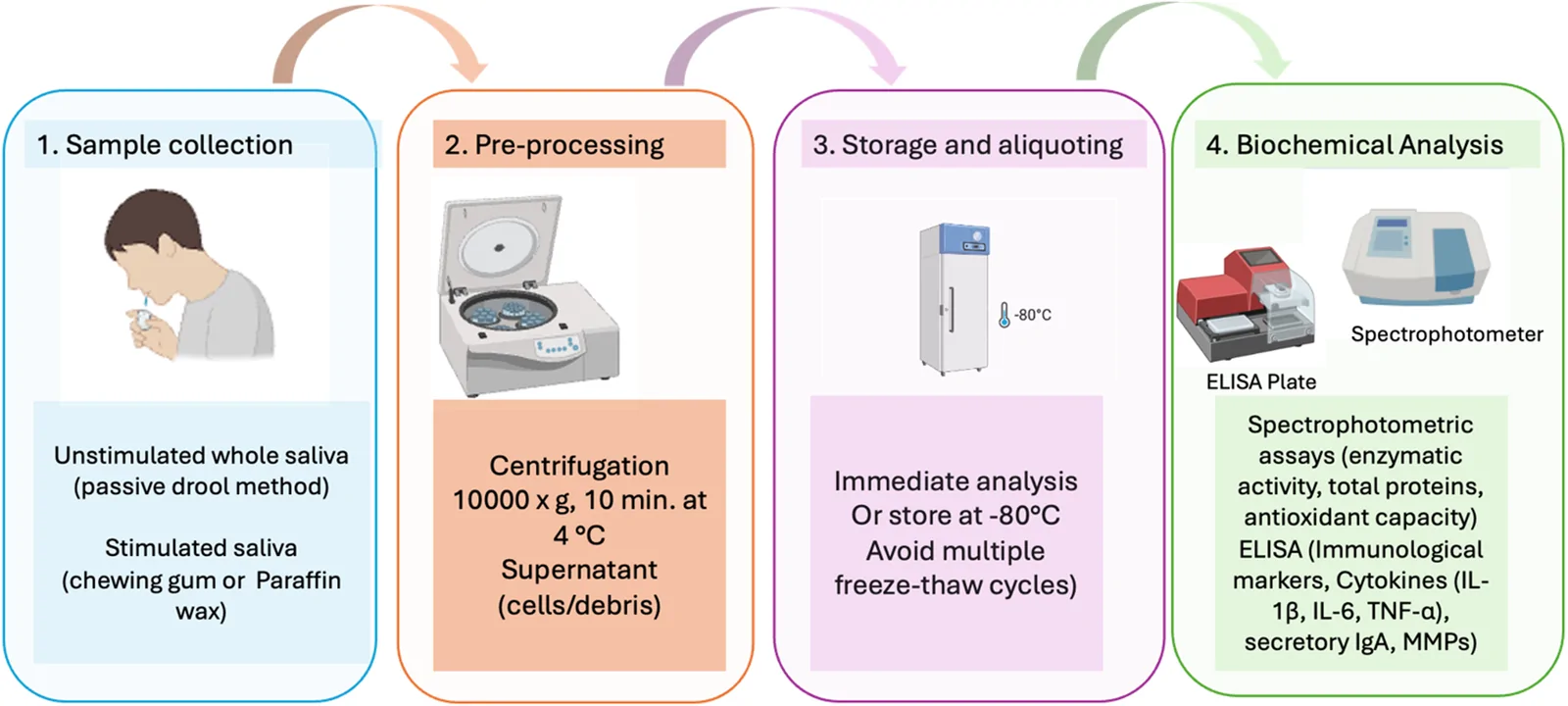
Standardized methodology for salivary sample processing. (1) Collection of unstimulated whole saliva via the passive drool technique or stimulated saliva using inert substrates. (2) Pre-processing through refrigerated centrifugation (10,000 × g for 10 min at 4 °C) to isolate the supernatant. (3) Aliquoting and storage at −80 °C, emphasizing the avoidance of multiple freeze-thaw cycles to maintain molecular integrity. (4) Quantitative biochemical and immunological analysis using spectrophotometry and ELISA platforms (ELISA, enzyme-linked immunosorbent assay; IgA, immunoglobulin A; IL-1β, interleukin 1 beta; IL-6, interleukin; MMPs: matrix metalloproteinases; TNF-α, tumor necrosis factor alpha).

Salivary biomarkers encompass a broad and heterogeneous spectrum of biochemical molecules, including proteins, enzymes, metabolites, nucleic acids, hormones, and inflammatory mediators, each reflecting distinct pathological processes within the oral environment. These biomarkers originate from salivary glands, oral epithelial cells, gingival crevicular fluid, and resident oral microorganisms. As a result, the salivary biomarker profile provides an integrated representation of local oral health status. A standardized overview of salivary sample processing, from collection to quantitative biochemical analysis, is presented in Figure 1.

A wide range of salivary biomarkers has been investigated in relation to oral diseases. In dental caries, alterations in salivary enzymes, microbial metabolites, immunological mediators, and buffering-related components have been associated with increased caries susceptibility and lesion progression. Periodontal diseases are commonly characterized by elevated levels of inflammatory cytokines, matrix metalloproteinases, and other proteolytic enzymes, reflecting ongoing tissue destruction and host immune responses. In oral mucosal disorders, including inflammatory and potentially malignant conditions, changes in oxidative stress markers, immune mediators, and epithelial-derived molecules have been correlated with disease severity and clinical evolution. Collectively, these biochemical signatures enhance understanding of oral disease mechanisms and provide opportunities for early diagnosis, risk stratification, and monitoring of disease progression.

Numerous clinical studies have demonstrated the relevance of biochemical salivary biomarkers in the diagnosis and monitoring of oral diseases, highlighting their close association with local inflammatory and pathological processes. In the context of dental caries and gingivitis, a case-control study incorporating machine learning validation, in which salivary cytokines such as interleukin-4 (IL-4), interleukin-13 (IL-13), IL-2 receptor alpha (IL-2RA), and Eotaxin/C-C motif chemokine ligand 11 were analyzed in healthy individuals, patients with gingivitis, and patients with deep carious lesions (115). Using a random forest model, the authors achieved accurate classification of dental caries in 37 out of 38 patients, demonstrating a strong correlation between salivary biomarker profiles and clinical diagnosis. These findings underline the high diagnostic potential of saliva-based immune signatures and suggest the involvement of JAK/STAT-related inflammatory pathways in caries development.

Salivary IL-2 levels were significantly increased in children with ALL following chemotherapy, suggesting its potential utility as a biomarker of treatment-related oral inflammation (116). Furthermore, elevated salivary levels of IL-1, IL-6, and Tumor necrosis factor alpha (TNF-α) in children who developed oral mucositis after chemotherapy, reinforcing the central role of inflammatory mediators in mucosal tissue damage (117). In adult patients, a progressive increase in buccal TNF-α concentrations following hematopoietic stem cell transplantation, proposing TNF-α as an early indicator of mucositis onset (118).

In dental caries, multiple studies have highlighted the association between salivary cytokine profiles and caries activity. A strong correlation between salivary IL-1β levels and *Streptococcus mutans* counts, suggesting that IL-1β may serve as a marker of microbial-driven caries activity (119). In a longitudinal study, a significant decrease in salivary IL-6 levels following dental treatment in children with caries, highlighting the potential of salivary biomarkers for monitoring treatment response (120).

Periodontal diseases have been consistently associated with elevated levels of inflammatory cytokines and matrix metalloproteinases in saliva. Significantly increased concentrations of IL-1β, MMP-8, and MMP-9 in patients with periodontitis compared to healthy controls, identifying these molecules as strong diagnostic biomarkers of periodontal tissue destruction (121). In an interventional study, a significant reduction in salivary IL-1β, IL-6, IL-8, and MMP-8 levels following periodontal therapy, supporting their utility in monitoring therapeutic outcomes (122). More recently, a cross-sectional metabolomic approach to evaluate salivary metabolic profiles across different stages of periodontitis (T1-T4). The authors identified progressive changes in metabolites such as glucose, 3-aminobutanoic acid, and methylmalonic acid, which correlated with disease severity, suggesting that salivary metabolomics may provide valuable biomarkers for disease staging and progression monitoring (123).

Salivary biomarkers have also shown promise in oral malignancies. Salivary levels of interleukin-10 (IL-10), TNF-α, tumour growth factor-beta, and vascular endothelial growth factor were correlated with tumor grade and viral status in patients with oropharyngeal squamous cell carcinoma, supporting their prognostic relevance (124). Similarly, a strong association between salivary IL-8 levels and metastatic potential in patients with tongue squamous cell carcinoma, highlighting its value as a prognostic biomarker (125).

Finally, a case-control study comparing salivary and serum biomarkers in recurrent aphthous stomatitis, oral lichen planus, and oral cancer (126). The authors reported significantly elevated salivary levels of IgA, cortisol, and lactate dehydrogenase in oral disease groups compared to controls, while serum biomarkers showed limited discriminatory power. These findings emphasize the superior sensitivity of saliva over serum in reflecting local oral pathological processes and reinforce the role of salivary biomarkers as non-invasive diagnostic tools in oral medicine (Table 2).

**Table 2:** Summary of representative studies on salivary biomarkers in oral.

Condition	Study design	Study population	Salivary biomarkers analyzed	Main findings	Diagnostic/prognostic relevance	References
Dental caries, gingivitis	Case-control with machine learning validation	Healthy controls (*n* = 18), gingivitis (*n* = 17), deep caries lesions (*n* = 38)	IL-4, IL-13, IL-2-RA, Eotaxin/CCL11	RF model accurately classified caries in 37/38 patients; strong correlation with clinical diagnosis	High diagnostic potential for non-invasive caries detection; suggests involvement of JAK/STAT signaling	(115)
Oral mucositis	Case-control study	78 children with ALL; 78 healthy controls	IL-2	Salivary IL-2 elevated after chemotherapy	Marker of treatment-related oral inflammation	(116)
	A randomized, controlled clinical trial	21 children with ALL; 21 healthy controls	IL-1, IL-6, TNF-α	Significant increase in salivary IL-6 and TNF-α at 96h	96 h is the ideal time for monitoring OM onset	(117)
	Longitudinal study	25 adults after HSCT	TNF-α	Buccal TNF-α increased after chemotherapy	Predictor of molecular events and pain severity in oral mucositis	(118)
Dental caries	Case-control study	74 children with caries; 34 controls	IL-1β, IL-1ra, IL-10	IL-1β correlated with *Streptococcus mutans* levels	Marker of caries activity	(119)
ECC	Longitudinal study	22 children with caries	IL-6	Significant reduction of salivary IL-6 after full mouth rehabilitation	Marker for treatment success and oral health-related quality of life	(120)
Periodontitis	Case-control study	30 periodontitis patients; 27 controls	CRP, IL-6, IL-8, IL-1β, IL-1ra, lactoferrin, MMP-8, MMP-9, PDGFBB, TNF-a, and VEGF	IL-1β, MMP-8, MMP-9 elevated; TNF-α decreased	Feasible for point-of-care diagnostic device (AUC 0.853)	(121)
	Interventional study	34 periodontitis patients; 20 controls	IL-1β, IL-6, IL-8, MMP-8	Biomarkers decreased after therapy	Salivary IL-1β and MMP-8 significantly reduced post-therapy	(122)
Periodontitis (stages T1-T4)	Cross-sectional metabolomic study	Healthy controls (*n* = 10); periodontitis patients T1-T4 (*n* *=* 106)	glucose, 3-aminobutanoic acid, methylmalonic acid	Progressive metabolic changes correlated with disease severity; distinct metabolite profiles across stages	Potential biomarkers for monitoring disease progression and identifying therapeutic targets	(123)
OSCC	Case-control study	78 OSCC patients; 40 controls	IL-10, TNF-α, TGF-β, VEGF	Cytokines correlated with tumor grade and viral status	Prognostic and stratification markers; Salivary VEGF levels were over 15 times higher in OSCC patients compared to controls (4,321 pg/mL vs. 280 pg/mL).	(124)
TSCC	Case-control study	18 TSCC patients; 56 controls	IL-1, IL-6, IL-8, VEGF, TNF-α	Elevation of IL-1α, IL-6, IL-8, VEGF, and TNF-α; significant correlation with endophytic growth and poor survival (10.4 months)	Prognostic tool for survival prediction and clinical stratification of endophytic vs. exophytic tumors	(125)
RAS, LP, OC	Case-control	RAS (*n* = 15), LP (*n* = 13), OC (*n* = 12), controls (*n* = 15)	IgA, cortisol, LDH	Significant increase in salivary IgA (*P* = .000), cortisol (*P* = .016), and LDH (*P* = .001) levels compared to the control group.	Salivary biomarkers showed higher sensitivity than serum markers for oral diseases; potential non-invasive diagnostic tools	(126)

ALL, acute lymphoblastic leukaemia; AUC, area under the curve; CCL11, C-C motif chemokine ligand 11; CRP, C-reactive protein; ECC, early childhood caries; IgA, immunoglobulin A; IL-1, Interleukin-1; IL-1α, Interleukin-1 alpha; IL-1β, Interleukin-1 beta; IL-1ra, interleukin-1 receptor antagonist; IL-2, Interleukin-2; IL-2-RA, interleukin-2 receptor alpha chain; IL-4, interleukin-4; IL-6, interleukin-6; IL-8, interleukin-8; IL-10, interleukin-10; IL-13, interleukin-13; GI, gingival index; HSCT, hematopoietic stem cell transplantation; JAK, Janus kinase; LDH, lactate dehydrogenase; LP, lichen planus; MMP-8, matrix metalloproteinase-8; MMP-9, matrix metalloproteinase-9; MTX, methotrexate; OC, oral cancer; OM, oral mucositis; OSCC, oropharyngeal squamous cell carcinoma; RAS, recurrent aphthous stomatitis; RF model, Random Forest model; STAT, signal transducer and activator of transcription proteins; TGF-β, tumour growth factor-beta; TNF-α, tumor necrosis factor alpha; TSCC, squamous cell carcinoma of the tongue; VEGF, vascular endothelial growth factor.

### 7.2 Diagnostic and prognostic applications

The diagnostic potential of salivary biomarkers lies in their capacity to capture pathological changes in real time while offering a minimally invasive and patient-friendly sampling method. Unlike conventional diagnostic approaches that rely on blood or tissue biopsies, saliva-based diagnostics enable repeated and stress-free collection, facilitating early disease detection, risk stratification, and continuous monitoring of disease dynamics (127). This feature is particularly advantageous in pediatric, elderly, and medically compromised populations, as well as in large-scale screening programs.

In the context of oral diseases, salivary biomarkers provide valuable insights into disease activity and progression. Quantitative and qualitative changes in inflammatory mediators, proteolytic enzymes, and oxidative stress markers can serve as indicators of active periodontal destruction, caries risk, or mucosal pathology (97). Furthermore, monitoring biomarker fluctuations over time allows for the evaluation of therapeutic efficacy and the personalization of treatment strategies. The integration of salivary biomarkers into clinical decision-making has the potential to shift oral healthcare toward a more predictive and preventive model.

Beyond oral health, growing evidence highlights the relevance of salivary biomarkers in the diagnosis and prognosis of systemic diseases, including metabolic disorders, autoimmune conditions, cardiovascular diseases, and malignancies. Saliva can reflect systemic biochemical alterations through the diffusion or active transport of molecules from the bloodstream, making it a valuable surrogate for systemic biomarkers. This dual diagnostic capability positions saliva as a unique medium for integrated oral-systemic health assessment.

The development of point-of-care diagnostic platforms and lab-on-a-chip technologies has further enhanced the clinical applicability of salivary biomarkers. These advances enable rapid, on-site analysis and real-time decision-making, reducing dependence on centralized laboratory infrastructure. Together, these diagnostic and prognostic applications underscore the growing importance of salivary biomarkers in modern precision medicine and preventive healthcare.

### 7.3 Advantages and limitations of saliva-based biomarkers

The use of saliva as a diagnostic medium offers several compelling advantages that have driven increasing interest in saliva-based biomarker research. Saliva collection is non-invasive, painless, and easily repeatable, allowing for frequent sampling without causing patient discomfort or stress (128). This characteristic is particularly beneficial for vulnerable populations, including children, elderly individuals, and patients with chronic conditions. In addition, saliva collection carries a low risk of infection transmission and does not require specialized personnel or equipment, making it well suited for large-scale screening programs and community-based studies. From a practical perspective, saliva-based diagnostics are generally cost-effective and logistically simpler than blood-based testing, facilitating broader clinical and epidemiological applications.

Despite these advantages, several limitations currently hinder the widespread clinical adoption of salivary biomarkers. Salivary composition is highly dynamic and can be influenced by numerous physiological and external factors, including salivary flow rate, circadian rhythms, hydration status, dietary intake, medication use, and oral hygiene practices. These variables contribute to inter- and intra-individual variability in biomarker concentrations, complicating data interpretation and comparison across studies. Furthermore, local oral conditions such as inflammation, infection, or tissue damage may alter biomarker levels independently of systemic disease, potentially confounding diagnostic accuracy. Addressing these methodological and biological challenges is essential for the successful translation of salivary biomarkers from research to clinical practice and for ensuring their reliability, validity, and clinical relevance.

## 8 Therapeutic and preventive implications

An improved understanding of the biochemical processes governing oral homeostasis and disease provides a foundation for the development of targeted therapeutic and preventive strategies. Interventions aimed at restoring or maintaining biochemical balance within the oral cavity have the potential to reduce disease risk, limit progression, and enhance long-term oral and systemic health outcomes.

### 8.1 Interventions targeting biochemical balance

Therapeutic approaches increasingly focus on modulating key biochemical parameters of the oral environment, including pH regulation, inflammatory mediator levels, and microbial metabolic activity. Strategies that enhance salivary flow and buffering capacity, such as salivary stimulants or substitutes, can help counteract acidogenic challenges and support enamel remineralization. Anti-inflammatory therapies aimed at reducing excessive host responses may also limit tissue destruction in periodontal and mucosal diseases.

In addition, the modulation of enzymatic activity and oxidative stress represents an important therapeutic avenue. Antioxidant-based interventions have been investigated for their potential to mitigate oxidative damage and restore redox balance in inflammatory oral conditions. By targeting underlying biochemical dysregulation rather than solely clinical symptoms, these approaches align with a more mechanistic and personalized model of oral healthcare.

### 8.2 Role of diet, oral hygiene, and probiotics

Dietary habits exert a profound and multifaceted influence on the biochemical environment of the oral cavity by shaping substrate availability for microbial metabolism and modulating host defense mechanisms. Frequent consumption of fermentable carbohydrates provides a continuous energy source for acidogenic microorganisms, leading to sustained acid production and prolonged decreases in local pH. In contrast, reducing both the quantity and frequency of sugar intake allows salivary buffering and remineralization processes to restore mineral balance at the tooth surface, thereby lowering the risk of dental caries. Diets enriched with essential micronutrients, such as calcium, phosphate, vitamins, and antioxidants, further contribute to maintaining enamel integrity, supporting epithelial health, and enhancing immune function within the oral cavity.

Effective oral hygiene practices are fundamental to controlling the biochemical consequences of microbial accumulation. Mechanical disruption of dental biofilms through brushing and interdental cleaning reduces microbial load and limits the development of mature, pathogenic biofilms characterized by harmful metabolic activity (129). Adjunctive chemical agents, including fluoride, antiseptics, and anti-inflammatory compounds, can further modulate microbial metabolism, enhance remineralization, and attenuate inflammatory responses. Together, these measures help preserve biochemical equilibrium and prevent shifts toward dysbiotic, disease-associated states.

In recent years, probiotics have gained increasing attention as a biologically based preventive strategy aimed at selectively modulating the oral microbiota. Probiotic microorganisms may compete with pathogenic species for adhesion sites and nutrients, produce antimicrobial substances, and influence local immune responses (63). By promoting a microbial community that supports biochemical stability rather than pathogenic metabolism, probiotics hold potential for reducing oral dysbiosis and enhancing oral homeostasis (130). Although further clinical evidence is needed to define optimal strains, dosages, and delivery methods, probiotic-based interventions represent a promising adjunct to conventional preventive approaches.

### 8.3 Emerging therapeutic perspectives

Emerging therapeutic strategies in oral healthcare increasingly capitalize on advances in molecular biology, biomaterials science, and precision medicine to address the biochemical mechanisms underlying oral diseases. Rather than relying solely on broad-spectrum antimicrobial approaches, current research emphasizes targeted interventions designed to selectively disrupt pathogenic pathways while preserving beneficial microbial functions and overall ecological balance. Targeted antimicrobial agents and enzyme inhibitors are being developed to interfere with specific metabolic activities, virulence factors, and biofilm-forming capabilities of pathogenic microorganisms, thereby reducing disease potential without promoting widespread microbial resistance.

Host-modulation therapies represent another promising avenue, focusing on regulating the host inflammatory and immune responses that contribute to tissue destruction. By modulating cytokine signaling, proteolytic enzyme activity, and oxidative stress pathways, these approaches aim to limit collateral tissue damage while maintaining essential defense mechanisms (131). Such strategies underscore a shift from symptom-oriented treatment toward mechanism-based intervention rooted in biochemical understanding.

Saliva-based diagnostics further allow for the integration of personalized medicine into oral healthcare by enabling the identification of individual biochemical risk profiles (132). Real-time monitoring of salivary biomarkers can inform treatment selection, optimize therapeutic timing, and evaluate treatment efficacy, thereby supporting tailored preventive and therapeutic strategies. This personalized approach aligns with broader trends in precision medicine and enhances the potential for early intervention.

In parallel, advances in biomaterials and drug delivery systems have led to the development of bioactive dental materials and controlled-release formulations capable of sustaining therapeutic effects within the oral cavity. These technologies enable localized, prolonged delivery of antimicrobial, anti-inflammatory, or remineralizing agents, improving treatment efficacy and patient compliance. Collectively, these emerging therapeutic perspectives reflect a paradigm shift toward preventive, mechanism-driven, and individualized oral healthcare that integrates biochemical insights into clinical practice and holds significant promise for improving long-term outcomes.

## 9 Future directions and research perspectives

Despite significant advances in understanding the biochemical processes of the oral cavity, substantial knowledge gaps remain that limit the full translation of research findings into clinical practice. Many biochemical mechanisms underlying oral health and disease are incompletely characterized, particularly with respect to the dynamic interactions between host factors, microbial metabolism, and environmental influences. In addition, variability in study design, analytical methodologies, and population characteristics complicates the interpretation and comparison of existing data. Addressing these gaps is essential for developing a more comprehensive and integrative model of oral biochemistry.

Current research has predominantly focused on isolated biochemical markers or individual microbial species, often overlooking the complexity of the oral ecosystem as an integrated biological system. The temporal dynamics of biochemical changes during disease initiation and progression remain poorly understood, as does the threshold at which physiological adaptations transition into pathological states (133). Furthermore, the mechanisms linking oral biochemical dysregulation to systemic diseases require further clarification, particularly in terms of causality vs. association. Greater emphasis on longitudinal studies and standardized analytical frameworks is needed to bridge these knowledge gaps.

The application of omics technologies offers unprecedented opportunities to advance oral biochemical research. Proteomics, metabolomics, transcriptomics, and microbiomics enable comprehensive profiling of salivary, microbial, and host-derived molecules, providing deeper insights into complex biochemical networks. Integration of multi-omics data can reveal previously unrecognized interactions and pathways involved in oral homeostasis and disease (134).

Advances in bioinformatics and systems biology further enhance the ability to interpret large datasets and identify clinically relevant biomarkers and therapeutic targets. As analytical sensitivity and computational tools continue to improve, omics-based approaches are expected to play an increasingly central role in the characterization of oral biochemical signatures and in the development of precision oral medicine.

Future research should prioritize interdisciplinary and translational approaches that integrate biochemical, microbiological, immunological, and clinical data. The development of standardized protocols for sample collection, analysis, and data reporting will be critical for improving reproducibility and facilitating cross-study comparisons. In addition, greater attention should be given to personalized risk assessment, leveraging salivary biomarkers and microbial profiles to tailor preventive and therapeutic interventions.

Emerging technologies, including artificial intelligence and machine learning, hold promise for analyzing complex oral biochemical datasets and predicting disease risk and treatment outcomes. Together, these future research directions underscore the need for a holistic, systems-based approach to oral biochemistry that not only deepens fundamental understanding but also supports the advancement of preventive, diagnostic, and therapeutic strategies in oral healthcare.

## 10 Strengths and limitations

A primary strength of this review lies in its comprehensive, integrative approach to oral biochemistry, bridging the gap between molecular interactions, advanced salivary diagnostics, and clinical dental pathology. By contextualizing the oral cavity as a dynamic biochemical ecosystem, this paper synthesizes vast areas of research, ranging from salivary proteomics and metabolomics to the physical chemistry of mineral homeostasis and enzyme-driven tissue degradation. Unlike highly narrow systematic reviews, this narrative format allows for a holistic conceptual framework that links multiple simultaneous pathogenic mechanisms, such as oxidative stress cascades, microbial dysbiosis, and matrix metalloproteinase activation, providing readers with a broader, interconnected understanding of oral health and disease.

Despite its comprehensive scope, several limitations must be acknowledged. Crucially, because this manuscript was conducted as a narrative review rather than a systematic review, it did not follow a strict, mathematically driven screening protocol (such as PRISMA). This methodological choice introduces an inherent risk of selection bias, as the identification and synthesis of the included literature relied on the authors' conceptual targeting rather than a rigid automated extraction.

Furthermore, the absence of a quantitative meta-analysis limits our ability to statistically standardize the conflicting data found in recent salivary biomarker and proteomic studies; variations in sample collection, storage methods, and patient demographics across the reviewed prior research could distort the perceived diagnostic accuracy of certain biochemical indicators. Consequently, while this review offers robust mechanistic insights, the clinical translations of the discussed biomarkers should be interpreted with caution, highlighting the urgent need for standardized multi-omic protocols in future oral biochemistry research.

## 11 Conclusions

This review highlights the oral cavity as a highly dynamic biochemical system in which host tissues, saliva, microbial communities, and environmental factors interact to maintain health or drive disease. The biochemical processes governing salivary function, microbial metabolism, immune responses, and tissue integrity are tightly interconnected, and disturbances in these processes underlie the pathogenesis of major oral diseases, including dental caries, periodontal disease, and oral mucosal disorders. Understanding these mechanisms provides a unifying framework for interpreting the transition from oral health to disease.

Maintaining oral biochemical homeostasis emerges as a central determinant of oral health. Balanced salivary composition, controlled microbial metabolism, effective buffering capacity, and regulated inflammatory responses are essential for preserving structural integrity and functional resilience within the oral cavity. Disruption of this equilibrium, often associated with microbial dysbiosis and biochemical imbalance, creates conditions that favor disease development and progression. Therapeutic and preventive strategies that support biochemical stability therefore represent a rational and effective approach to oral healthcare.

From a clinical perspective, advances in oral biochemistry have significant implications for diagnosis, prevention, and treatment. The identification of salivary biomarkers offers promising opportunities for non-invasive disease detection, risk assessment, and monitoring of therapeutic outcomes, while emerging targeted and personalized interventions reflect a shift toward mechanism-driven clinical practice. Integrating biochemical insights into routine dental care has the potential to enhance preventive strategies, improve patient outcomes, and strengthen the link between oral and systemic health.

## References

[1] Hou J Xiang J Li D Liu X Pan W. Gut microbial response to host metabolic phenotypes. Front Nutr. (2022) 9:1019430. 10.3389/fnut.2022.1019430 36419554 PMC9676441

[2] Fábián TK Hermann P Beck A Fejérdy P Fábián G. Salivary defense proteins: their network and role in innate and acquired oral immunity. Int J Mol Sci. (2012) 13:4295–320. 10.3390/ijms13044295 22605979 PMC3344215

[3] Rajasekaran JJ Krishnamurthy HK Bosco J Jayaraman V Krishna K Wang T. Oral microbiome: a review of its impact on oral and systemic health. Microorganisms. (2024) 12:1797. 10.3390/microorganisms12091797 39338471 PMC11434369

[4] Chibly A Aure M Patel V Hoffman MP. Salivary gland function, development, and regeneration. Physiol Rev. (2022) 102:1495–552. 10.1152/physrev.00015.2021 35343828 PMC9126227

[5] Sedghi L DiMassa V Harrington A Lynch SV Kapila YL. The oral microbiome: role of key organisms and Complex networks in oral health and disease. Periodontol 2000. (2021) 87:107–31. 10.1111/prd.12393 34463991 PMC8457218

[6] Marya A Rokaya D Heboyan A De Oliveira Fernandes GV. Biomolecular and biochemical aspects of the oral cavity. Molecules. (2022) 27:8676. 10.3390/molecules27248676 36557808 PMC9782879

[7] Liu S Liu J Wang Y Deng F Deng Z. Oxidative stress: signaling pathways, biological functions, and disease. MedComm (Beijing). (2025) 6:e70268. 10.1002/mco2.70268 PMC12209598 40599237

[8] Spatafora G Li Y He X Cowan A Tanner ACR. The evolving microbiome of dental caries. Microorganisms. (2024) 12:121. 10.3390/microorganisms12010121 38257948 PMC10819217

[9] Sánchez-Pascuala A De Lorenzo V Nikel PI. Refactoring the embden–meyerhof–parnas pathway as a whole of portable GlucoBricks for implantation of glycolytic modules in gram-negative Bacteria. ACS Synth Biol. (2017) 6:793–805. 10.1021/ACSSYNBIO.6B00230 28121421 PMC5440799

[10] Franco C Patricia HR Timo S Claudia B Marcela H. Matrix metalloproteinases as regulators of periodontal inflammation. Int J Mol Sci. (2017) 18:440. 10.3390/IJMS18020440 28218665 PMC5343974

[11] Hasturk H Kantarci A Van Dyke TE. Oral inflammatory diseases and systemic inflammation: role of the macrophage. Front Immunol. (2012) 3:118. 10.3389/fimmu.2012.00118 22623923 PMC3353263

[12] Sáenz-Ravello G Hernández M Baeza M Hernández-Ríos P. The role of oral biomarkers in the assessment of noncommunicable diseases. Diagnostics. (2025) 15:78. 10.3390/diagnostics15010078 PMC11719684 39795606

[13] Mendes JJ. Advancing the understanding of oral health through multidisciplinary and translational perspectives insights from a special issue of the journal of clinical medicine. J Clin Med. (2025) 14:6050. 10.3390/jcm14176050 40943810 PMC12428913

[14] Nagakubo D Kaibori Y. Oral microbiota: the influences and interactions of saliva, IgA, and dietary factors in health and disease. Microorganisms. (2023) 11:2307. 10.3390/MICROORGANISMS11092307 37764151 PMC10535076

[15] Liu Y Yu S Wang X Ye D Aili M Fu X. Integrated salivary microbiome and metabolome profiling reveals ecological and functional alterations in severe early childhood caries. J Transl Med. (2026) 24:53. 10.1186/s12967-025-07541-9 PMC12798063 41361886

[16] De Souza TR Zancope BR De Sousa ET Parisotto TM Marques MR Dos Santos MN. Sucrose rinse modulates the salivary behavior of carbonic anhydrase VI and its buffering capacity: a longitudinal study in 4 to 6.5-year-old children. PeerJ. (2024) 12:e17429. 10.7717/PEERJ.17429/SUPP-2 38827285 PMC11144396

[17] Kalantzis I Natsi PD Koutsoukos PG. Crystal growth and dissolution of hydroxyapatite: the role of ascorbic acid. Crystals (Basel). (2025) 15:790. 10.3390/CRYST15090790

[18] Tani H Uchida H Suzuki T Shibuya Y Shimanuki H Watanabe K. Dental conditions in inpatients with schizophrenia: a large-scale multi-site survey. BMC Oral Health. (2012) 12:32. 10.1186/1472-6831-12-32 22901247 PMC3466126

[19] Ruhl S. The scientific exploration of saliva in the post-proteomic era: from database back to basic function. Expert Rev Proteomics. (2012) 9:85–96. 10.1586/epr.11.80 22292826 PMC3289089

[20] Kagami H Hiramatsu Y Hishida S Okazaki Y Horie K Oda Y. Salivary growth factors in health and disease. Adv Dent Res. (2000) 14:99–102. 10.1177/08959374000140011601 11842932

[21] Subbarao K Nattuthurai G Sundararajan S Sujith I Joseph J Syedshah Y. Gingival crevicular fluid: an overview. J Pharm Bioallied Sci. (2019) 11(Suppl 2):S135–S139. 10.4103/JPBS.JPBS_56_19. PMID: . 31198325 PMC6555362

[22] Lamont RJ Koo H Hajishengallis G. The oral microbiota: dynamic communities and host interactions. Nat Rev Microbiol. (2018) 16:745–59. 10.1038/s41579-018-0089-x 30301974 PMC6278837

[23] Baker JL Mark Welch JL Kauffman KM McLean JS He X. The oral microbiome: diversity, biogeography and human health. Nat Rev Microbiol. (2024) 22:89–104. 10.1038/s41579-023-00963-6 37700024 PMC11084736

[24] Han Z Zhao L Hu Q Hung I Liu C Liu S. Gut microbiota-mediated modulation of host amino acid availability and metabolism. Gut Microbes. (2025) 17:2552345. 10.1080/19490976.2025.2552345 40878016 PMC12407847

[25] Rajendra RE Srikanth S Kiranmayi M Swathi SP Dutta LD Kumar A. Evaluation of flow rate, PH, and buffering capacity of saliva in children with caries, fluorosis, and caries with fluorosis. Int J Clin Pediatr Dent. (2023) 16:587–90. 10.5005/jp-journals-10005-2645 37731792 PMC10507300

[26] Antoniadou M Varzakas T. Oral cellular homeostasis and occupational wellbeing in healthcare professionals under the lens of salivary, immune, and microbiome mechanisms. Cells. (2026) 15:406. 10.3390/cells15050406 41827840 PMC12984415

[27] Lee YH Chung SW Auh QS Hong SJ Lee YA Jung J. Progress in oral microbiome related to oral and systemic diseases: an update. Diagnostics (Basel). (2021) 11:1283. 10.3390/diagnostics11071283 34359364 PMC8306157

[28] de Molon RS Vernal R Oliveira GE Steffens JP Ervolino E Theodoro LH. Inflammatory bone loss and signaling pathways in periodontitis: mechanistic insights and emerging therapeutic strategies. Bone Res. (2026) 14:1. 10.1038/s41413-025-00478-1 41484074 PMC12764867

[29] Lee YS Olefsky J. Chronic tissue inflammation and metabolic disease. Genes Dev. (2021) 35:307–28. 10.1101/GAD.346312.120 33649162 PMC7919414

[30] Zhang S Paul S Kundu P. NF-*Κ*B regulation by gut microbiota decides homeostasis or disease outcome during ageing. Front Cell Dev Biol. (2022) 10:874940. 10.3389/FCELL.2022.874940 35846362 PMC9285657

[31] Chen Y-S Tian H-X Rong D-C Wang L Chen S Zeng J. ROS homeostasis in cell fate, pathophysiology, and therapeutic interventions. Mol Biomed. (2025) 6:89. 10.1186/S43556-025-00338-8 41162811 PMC12572517

[32] Shah SD Gupta S Pamu PK Shukla JP Nayak S Chatterjee K. The role of salivary diagnostics in early detection of systemic and oral diseases: a comprehensive review. Cureus. (2025) 17:e100313. 10.7759/cureus.100313 41613689 PMC12848634

[33] Okuyama K Yanamoto S. Saliva in balancing oral and systemic health, oral cancer, and beyond: a narrative review. Cancers (Basel). (2024) 16:4276. 10.3390/cancers16244276 39766175 PMC11674559

[34] Matsuoka M Soria SA Pires JR Sant’Ana ACP Freire M. Natural and induced immune responses in oral cavity and saliva. BMC Immunol. (2025) 26:34. 10.1186/s12865-025-00713-8 40251519 PMC12007159

[35] Schwerdt G Schulz MC Kopf M Mildenberger S Reime S Gekle M. Physiological regulation of oral Saliva ion composition and flow rate are not coupled in healthy humans-partial revision of our current knowledge required. Pflugers Arch. (2025) 477:55–65. 10.1007/s00424-024-03025-9 39354192 PMC11711875

[36] Dawes C. Circadian rhythms in human salivary flow rate and composition. J Physiol. (1972) 220:529–45. 10.1113/jphysiol.1972.sp009721 5016036 PMC1331668

[37] Sadashivappa Pateel DG Gunjal S Dutta S. Association of salivary statherin, calcium, and proline-rich proteins: a potential predictive marker of dental caries. Contemp Clin Dent. (2022) 13:84–9. 10.4103/ccd.ccd_859_20 35466299 PMC9030311

[38] Peyrot des Gachons C Breslin PAS. Salivary amylase: digestion and metabolic syndrome. Curr Diab Rep. (2016) 16:102. 10.1007/s11892-016-0794-7 27640169 PMC6825871

[39] Dumitru CN Mariana L Budacu CC Mitea G Radu MD Dumitru AO. Balancing the oral redox state: endogenous and exogenous sources of reactive oxygen species and the antioxidant role of Lamiaceae and Asteraceae. Dentistry Journal. (2025) 13:222. 10.3390/dj13050222 40422642 PMC12110304

[40] Sedek EM Holiel AA. Next-generation strategies for enamel repair and regeneration: advances in biomaterials and translational challenges. Tissue Eng Regen Med. (2025) 22:771–89. 10.1007/s13770-025-00725-w 40347432 PMC12297108

[41] Wang L Jiang S Zhou J Gholipourmalekabadi M Cao Y Lin K. From hard tissues to beyond: progress and challenges of strontium-containing biomaterials in regenerative medicine applications. Bioact Mater. (2025) 49:85–120. 10.1016/j.bioactmat.2025.02.039 40124596 PMC11928986

[42] Vila T Rizk AM Sultan AS Jabra-Rizk MA. The power of saliva: antimicrobial and beyond. PLoS Pathog. (2019) 15:e1008058. 10.1371/journal.ppat.1008058 31725797 PMC6855406

[43] Bechir F Pacurar M Tohati A Bataga SM. Comparative study of salivary PH, buffer capacity, and flow in patients with and without gastroesophageal reflux disease. Int J Environ Res Public Health. (2022) 19:201. 10.3390/IJERPH19010201 PMC8750732 35010461

[44] Anderson LA Orchardson R. The effect of chewing bicarbonate-containing gum on salivary flow rate and PH in humans. Arch Oral Biol. (2003) 48:201–4. 10.1016/S0003-9969(02)00214-5 12648557

[45] Farooq I Bugshan A. The role of salivary contents and modern technologies in the remineralization of dental enamel: a narrative review [version 3; peer review: 3 approved]. F1000Res. (2020) 9:1–14. 10.12688/F1000RESEARCH.22499.3 PMC7076334 32201577

[46] Neel EAA Aljabo A Strange A Ibrahim S Coathup M Young AM. Demineralization–remineralization dynamics in teeth and bone. Int J Nanomedicine. (2016) 11:4743–63. 10.2147/IJN.S107624 27695330 PMC5034904

[47] Ahmad L Aljoujou AA Nadra R Mashlah AM Al Beesh FA Alyafi A. The association between dental caries and salivary buffering capacity in Syrian patients diagnosed with sickle cell disease. Cureus. (2024) 16:e64887. 10.7759/cureus.64887 39156342 PMC11330576

[48] Iorgulescu, G. Saliva between Normal and Pathological. Important factors in determining systemic and oral health. J Med Life. (2009) 2(3):303–7. https://pmc.ncbi.nlm.nih.gov/articles/PMC5052503/. PMID: . 20112475 PMC5052503

[49] Berg ICH Rutland MW Arnebrant T. Lubricating properties of the initial salivary pellicle–an AFM study. Biofouling. (2003) 19:365–9. 10.1080/08927010310001618571 14768465

[50] Hannig M Hannig C. The pellicle and erosion. Monogr Oral Sci. (2014) 25:206–14. 10.1159/000360376 24993268

[51] Martin LE Gutierrez VA Torregrossa AM. The role of saliva in taste and food intake. Physiol Behav. (2023) 262:114109. 10.1016/j.physbeh.2023.114109 36740133 PMC10246345

[52] Philip N. State of the art enamel remineralization systems: the next frontier in caries management. Caries Res. (2019) 53:284–95. 10.1159/000493031 30296788 PMC6518861

[53] Buck F Bugter J Yorukoglu G Kazemzadeh Dastjerd M Winkler TE. Personalized models of biological barriers and their diseases: recent progress with organs-on-chips. Adv Biol. (2026) 10:e00536. 10.1002/ADBI.202500536 PMC12893407 41671052

[54] Ingendoh-Tsakmakidis A Mikolai C Winkel A Szafrański SP Falk CS Rossi A. Commensal and pathogenic biofilms differently modulate peri-implant oral mucosa in an organotypic model. Cell Microbiol. (2019) 21:e13078. 10.1111/CMI.13078 31270923 PMC6771885

[55] Juarez VM Montalbine AN Singh A. Microbiome as an immune regulator in health, disease, and therapeutics. Adv Drug Deliv Rev. (2022) 188:114400. 10.1016/j.addr.2022.114400 35718251 PMC10751508

[56] Keith JW Pamer EG. Enlisting commensal microbes to resist antibiotic-resistant pathogens. J Exp Med. (2019) 216:10–9. 10.1084/jem.20180399 30309968 PMC6314519

[57] Aggarwal N Kitano S Puah GRY Kittelmann S Hwang IY Chang MW. Microbiome and human health: current understanding, engineering, and enabling technologies. Chem Rev. (2023) 123:31–72. 10.1021/acs.chemrev.2c00431 36317983 PMC9837825

[58] Li X Liu Y Yang X Li C Song Z. The oral microbiota: community composition, influencing factors, pathogenesis, and interventions. Front Microbiol. (2022) 13:895537. 10.3389/fmicb.2022.895537 35572634 PMC9100676

[59] Mancera Azamar KM Sudi SD Mohammadalizadeh Z Coffin C Parker IK Porras AM. Innovative engineering approaches to model host-microbiome interactions *in vitro*. Adv Drug Deliv Rev. (2025) 226:115677. 10.1016/j.addr.2025.115677 40915424 PMC12646538

[60] Azzolino D Carnevale-Schianca M Santacroce L Colella M Felicetti A Terranova L. The oral-gut microbiota axis across the lifespan: new insights on a forgotten interaction. Nutrients. (2025) 17:2538. 10.3390/nu17152538 40806122 PMC12349001

[61] Aruni AW Dou Y Mishra A Fletcher HM. The biofilm community-rebels with a cause. Curr Oral Health Rep. (2015) 2:48–56. 10.1007/s40496-014-0044-5 26120510 PMC4478205

[62] Kashyap B Padala SR Kaur G Kullaa A. Candida Albicans induces oral microbial dysbiosis and promotes oral diseases. Microorganisms. (2024) 12:2138. 10.3390/microorganisms12112138 39597528 PMC11596246

[63] Banar M Rokaya D Azizian R Khurshid Z Banakar M. Oral bacteriophages: metagenomic clues to interpret microbiomes. PeerJ. (2024) 12:e16947. 10.7717/peerj.16947 38406289 PMC10885796

[64] Bostanghadiri N Kouhzad M Taki E Elahi Z Khoshbayan A Navidifar T. Oral microbiota and metabolites: key players in oral health and disorder, and microbiota-based therapies. Front Microbiol. (2024) 15:1431785. 10.3389/fmicb.2024.1431785 39228377 PMC11368800

[65] Atasoy M Ordóñez AÁ Cenian A Djukić-Vuković A Lund PA Ozogul F. Exploitation of microbial activities at low PH to enhance planetary health. FEMS Microbiol Rev. (2024) 48:fuad062. 10.1093/femsre/fuad062 37985709 PMC10963064

[66] Weiner ID Mitch WE Sands JM. Urea and ammonia metabolism and the control of renal nitrogen excretion. Clin J Am Soc Nephrol. (2015) 10:1444–58. 10.2215/CJN.10311013 25078422 PMC4527031

[67] Rowland I Gibson G Heinken A Scott K Swann J Thiele I. Gut microbiota functions: metabolism of nutrients and other food components. Eur J Nutr. (2018) 57:1–24. 10.1007/s00394-017-1445-8 28393285 PMC5847071

[68] Fusco W Lorenzo MB Cintoni M Porcari S Rinninella E Kaitsas F. Short-chain fatty-acid-producing bacteria: key components of the human gut microbiota. Nutrients. (2023) 15:2211. 10.3390/nu15092211 37432351 PMC10180739

[69] Cebi M Yilmaz Y. Epithelial barrier hypothesis in the context of nutrition, microbial dysbiosis, and immune dysregulation in metabolic dysfunction-associated steatotic liver. Front Immunol. (2025) 16:1575770. 10.3389/fimmu.2025.1575770 40438102 PMC12116361

[70] Marsh PD. Dental plaque as a biofilm and a microbial community—implications for health and disease. BMC Oral Health. (2006) 6(Suppl 1):S14. 10.1186/1472-6831-6-S1-S14 16934115 PMC2147593

[71] Deari S Gothwal M Gränicher K Thurnheer T Attin T Karygianni L. The effect of pellicle on biofilm formation in a supragingival biofilm model. Clin Exp Dent Res. (2025) 11:e70276. 10.1002/cre2.70276 41457737 PMC12745897

[72] Milani C Duranti S Bottacini F Casey E Turroni F Mahony J. The first microbial colonizers of the human gut: composition, activities, and health implications of the infant gut microbiota. Microbiol Mol Biol Rev. (2017) 81:e00036–17. 10.1128/mmbr.00036-17 29118049 PMC5706746

[73] Peters BM Jabra-Rizk MA O’May GA William Costerton J Shirtliff ME. Polymicrobial interactions: impact on pathogenesis and human disease. Clin Microbiol Rev. (2012) 25:193–213. 10.1128/CMR.00013-11 22232376 PMC3255964

[74] Wang X Liu M Yu C Li J Zhou X. Biofilm formation: mechanistic insights and therapeutic targets. Mol Biomed. (2023) 4:49. 10.1186/s43556-023-00164-w 38097907 PMC10721784

[75] Almatroudi A. Biofilm resilience: molecular mechanisms driving antibiotic resistance in clinical contexts. Biology (Basel). (2025) 14:165. 10.3390/biology14020165 40001933 PMC11852148

[76] Sauer K Stoodley P Goeres DM Hall-Stoodley L Burmølle M Stewart PS. The biofilm life cycle: expanding the conceptual model of biofilm formation. Nat Rev Microbiol. (2022) 20:608–20. 10.1038/s41579-022-00767-0 35922483 PMC9841534

[77] Shrestha HK Appidi MR Villalobos Solis MI Wang J Carper DL Burdick L. Metaproteomics reveals insights into microbial structure, interactions, and dynamic regulation in defined communities as they respond to environmental disturbance. BMC Microbiol. (2021) 21:308. 10.1186/s12866-021-02370-4 34749649 PMC8574000

[78] Sultan S El-Mowafy M Elgaml A Ahmed TAE Hassan H Mottawea W. Metabolic influences of gut microbiota dysbiosis on inflammatory bowel disease. Front Physiol. (2021) 12:715506. 10.3389/fphys.2021.715506 34646151 PMC8502967

[79] Hoare A Marsh PD Diaz PI. Ecological therapeutic opportunities for oral diseases. Microbiol Spectr. (2017) 5:10.1128/microbiolspec.bad-0006-2016. 10.1128/microbiolspec.bad-0006-2016 28840820 PMC5573124

[80] Pokharel K Dawadi BR Shrestha LB. Role of biofilm in bacterial infection and antimicrobial resistance. JNMA J Nepal Med Assoc. (2022) 60:836–40. 10.31729/jnma.7580 36705135 PMC9794942

[81] Ray RR. Biofilm architecture and dynamics of the oral ecosystem. Biotechnologia. (2024) 105:395–402. 10.5114/bta.2024.145259 39844868 PMC11748217

[82] Flemming J Hannig C Hannig M. Caries management-the role of surface interactions in de- and remineralization-processes. J Clin Med. (2022) 11:7044. 10.3390/jcm11237044 36498618 PMC9737279

[83] Paes Leme AF Koo H Bellato CM Bedi G Cury JA. The role of sucrose in cariogenic dental biofilm formation–new insight. J Dent Res. (2006) 85:878–87. 10.1177/154405910608501002 16998125 PMC2257872

[84] Lemos JA Palmer SR Zeng L Wen ZT Kajfasz JK Freires IA. The biology of Streptococcus Mutans. Microbiol Spectr. (2019) 7:10.1128/microbiolspec.gpp3-0051-2018. 10.1128/microbiolspec.gpp3-0051-2018 30657107 PMC6615571

[85] Kawada-Matsuo M Oogai Y Komatsuzawa H. Sugar allocation to metabolic pathways is tightly regulated and affects the virulence of Streptococcus Mutans. Genes (Basel). (2017) 8:11. 10.3390/GENES8010011 PMC5295006 28036052

[86] Safavi MS Walsh FC Surmeneva MA Surmenev RA Khalil-Allafi J. Electrodeposited hydroxyapatite-based biocoatings: recent progress and future challenges. Coatings. (2021) 11:110. 10.3390/COATINGS11010110

[87] Loewe MF Doll-Nikutta K Stiesch M Schwestka-Polly R. Biofilm volume and acidification within initial biofilms Formed *in situ* on buccally and palatally exposed bracket material. J Orofac Orthop. (2025) 86:259–70. 10.1007/s00056-024-00515-4 38409443 PMC12181129

[88] Ersen MC Çelik ZC Oztas M Sahin M Tagtekin D Yanikoglu F. Impact of demineralization time on enamel microhardness reduction and lesion depth: an *in vitro* study. Cureus. (2025) 17:e79441. 10.7759/cureus.79441 40130153 PMC11931587

[89] Enax J Fandrich P Schulze zur Wiesche E Epple M. The remineralization of enamel from saliva: a chemical perspective. Dent J. (2024) 12:339. 10.3390/dj12110339 PMC11592461 39590389

[90] Roberts WE Mangum JE Schneider PM. Pathophysiology of demineralization, part II: enamel white spots, cavitated caries, and bone infection. Curr Osteoporos Rep. (2022) 20:106–19. 10.1007/s11914-022-00723-0 35156182 PMC8930953

[91] Dobrota CT Florea A-D Racz C-P Tomoaia G Soritau O Avram A. Dynamics of dental enamel surface remineralization under the action of toothpastes with substituted hydroxyapatite and birch extract. Materials (Basel). (2024) 17:2038. 10.3390/MA17092038 38730845 PMC11084803

[92] Mareddy AR Reddy VN Done V Rehaman T Gadekar T Raj M. Comparative evaluation of three remineralization agents—bioactive glass, nanohydroxyapatite and casein phosphopeptide-amorphous calcium phosphate fluoride-based slurry on enamel erosion of primary teeth: an *in vitro* study. Int J Clin Pediatr Dent. (2025) 18:425–30. 10.5005/JP-JOURNALS-10005-3032 40469815 PMC12131052

[93] Yang Q Li F Ye Y Zhang X. Antimicrobial, remineralization, and infiltration: advanced strategies for interrupting dental caries. Medical Review. (2025) 5:87–116. 10.1515/MR-2024-0035 40224367 PMC11987509

[94] Tassery H Sauro S Slimani A. Bioactive and ion-releasing materials in minimum intervention dentistry: a clinical pathway from prevention to restorative treatment. Frontiers in Dental Medicine. (2026) 7:1739208. 10.3389/FDMED.2026.1739208/TEXT 42027602 PMC13099902

[95] Belluz M Longhi EV. Periodontal disease. In: Managing Psychosexual Consequences in Chronic Diseases. Cham: Springer International Publishing (2023). p. 329–36. 10.1007/978-3-031-31307-3_27

[96] Mehrnia N Van Dyke TE. Microbial dysbiosis and immune dysregulation in periodontitis and peri-implantitis. Front Cell Infect Microbiol. (2026) 15:1678163. 10.3389/fcimb.2025.1678163 41574302 PMC12819797

[97] Martínez-García M Hernández-Lemus E. Periodontal inflammation and systemic diseases: an overview. Front Physiol. (2021) 12:709438. 10.3389/fphys.2021.709438 34776994 PMC8578868

[98] Shahoumi LA Saleh MHA Meghil MM. Virulence factors of the periodontal pathogens: tools to evade the host immune response and promote carcinogenesis. Microorganisms. (2023) 11:115. 10.3390/microorganisms11010115 36677408 PMC9860638

[99] Chen L Deng H Cui H Fang J Zuo Z Deng J. Inflammatory responses and inflammation-associated diseases in organs. Oncotarget. (2018) 9:7204–18. 10.18632/oncotarget.23208 29467962 PMC5805548

[100] Pizzino G Irrera N Cucinotta M Pallio G Mannino F Arcoraci V. Oxidative stress: harms and benefits for human health. Oxid Med Cell Longev. (2017) 2017:8416763. 10.1155/2017/8416763 28819546 PMC5551541

[101] Radzki D Negri A Kusiak A Obuchowski M. Matrix metalloproteinases in the periodontium-vital in tissue turnover and unfortunate in periodontitis. Int J Mol Sci. (2024) 25:2763. 10.3390/ijms25052763 38474009 PMC10932118

[102] Herszényi L Hritz I Lakatos G Varga MZ Tulassay Z. The behavior of matrix metalloproteinases and their inhibitors in colorectal cancer. Int J Mol Sci. (2012) 13:13240–63. 10.3390/ijms131013240 23202950 PMC3497324

[103] Rovai ES Holzhausen M. The role of proteinase-activated receptors 1 and 2 in the regulation of periodontal tissue metabolism and disease. J Immunol Res. (2017) 2017:1–13. 10.1155/2017/5193572 PMC5414592 28503577

[104] Marshall NC Finlay BB Overall CM. Sharpening host defenses during infection: proteases cut to the chase. Mol Cell Proteomics. (2017) 16:S161–71. 10.1074/mcp.O116.066456 28179412 PMC5393396

[105] Checchi V Maravic T Bellini P Generali L Consolo U Breschi L. The role of matrix metalloproteinases in periodontal disease. Int J Environ Res Public Health. (2020) 17:4923. 10.3390/IJERPH17144923 32650590 PMC7399864

[106] Li N Collyer CA. Gingipains from Porphyromonas Gingivalis—complex domain structures confer diverse functions. Eur J Microbiol Immunol (Bp). (2011) 1:41–58. 10.1556/EUJMI.1.2011.1.7 24466435 PMC3894813

[107] Korkmaz B Horwitz MS Jenne DE Gauthier F. Neutrophil elastase, proteinase 3, and cathepsin G as therapeutic targets in human diseases. Pharmacol Rev. (2010) 62:726–59. 10.1124/PR.110.002733 21079042 PMC2993259

[108] Sun W Fan B Qin X Zhang X Zhang P Zhang Y. Synergistic ROS/enzyme dual-responsive oral drug delivery system: a novel multi-mechanistic platform for spatiotemporal control and overcoming drug resistance in colorectal cancer therapy. Mater Today Bio. (2025) 33:101920. 10.1016/J.MTBIO.2025.101920 40528838 PMC12173668

[109] Tam K Torres VJ. Staphylococcus Aureus secreted toxins and extracellular enzymes. Microbiol Spectr. (2019) 7:10.1128/microbiolspec.gpp3-0039-2018. 10.1128/MICROBIOLSPEC.GPP3-0039-2018 30873936 PMC6422052

[110] Choi SY. Understanding of oral potentially malignant disorders and epithelial dysplasia among oral and maxillofacial surgeons. J Korean Assoc Oral Maxillofac Surg. (2023) 49:309–10. 10.5125/jkaoms.2023.49.6.309 38155083 PMC10761314

[111] Song X Liang H Nan F Chen W Li J He L. Autoimmune diseases: molecular pathogenesis and therapeutic targets. MedComm (Beijing). (2025) 6:e70262. 10.1002/mco2.70262 PMC12171081 40529617

[112] Glavina A Martić D Perko MA Mešin Delić D Tadin A Lešić S. The oral microbiome and systemic health: current insights into the mouth–body connection. Life. (2026) 16:294. 10.3390/life16020294 41752930 PMC12941995

[113] Sardaro N Della Vella F Incalza MA Stasio DDI Lucchese A Contaldo M. Oxidative stress and oral mucosal diseases: an overview. In Vivo. (2019) 33:289–96. 10.21873/invivo.11474 30804105 PMC6506298

[114] Marcotte H Lavoie MC. Oral microbial ecology and the role of salivary immunoglobulin A. Microbiol Mol Biol Rev. (1998) 62:71–109. 10.1128/mmbr.62.1.71-109.1998 9529888 PMC98907

[115] Paqué PN Herz C Wiedemeier DB Mitsakakis K Attin T Bao K. Salivary biomarkers for dental caries detection and personalized monitoring. J Pers Med. (2021) 11:235. 10.3390/jpm11030235 33806927 PMC8004821

[116] Pels E. Comparison of saliva interleukin-2 concentration to the condition of gums in children with acute lymphoblastic leukaemia during anti-tumour treatment. Cancer Chemother Pharmacol. (2015) 76:205–10. 10.1007/s00280-015-2750-7 25976216 PMC4485694

[117] Morales-Rojas T Viera N Morón-Medina A Álvarez CJ Álvarez A. Proinflammatory cytokines during the initial phase of oral mucositis in patients with acute lymphoblastic leukaemia. Int J Paediatr Dent. (2012) 22:191–6. 10.1111/j.1365-263X.2011.01175.x 21919984

[118] FalI-Dickson JM Ramsay ES Castro K Woltz P Sportés C. Oral mucositis-related oropharyngeal pain and correlative tumor necrosis factor-alpha expression in adult oncology patients undergoing hematopoietic stem cell transplantation. Clin Ther. (2007) 29(Suppl):2547–61. 10.1016/j.clinthera.2007.12.004 18164921

[119] Cogulu D Onay H Ozdemir Y Aslan GI Ozkinay F Kutukculer N. Associations of interleukin (IL)-1β, IL-1 receptor antagonist, and IL-10 with dental caries. J Oral Sci. (2015) 57:31–6. 10.2334/josnusd.57.31 25807906

[120] Menon MM Balagopal RV Sajitha K Parvathy K Sangeetha GB Arun XM. Evaluation of salivary interleukin-6 in children with early childhood caries after treatment. Contemp Clin Dent. (2016) 7:198. 10.4103/0976-237X.183059 27307667 PMC4906863

[121] Wu YC Ning L Tu YK Huang CP Huang NT Chen YF. Salivary biomarker combination prediction model for the diagnosis of periodontitis in a Taiwanese population. J Formos Med Assoc. (2018) 117:841–8. 10.1016/j.jfma.2017.10.004 29129647

[122] Lee CH Chen YW Tu YK Wu YC Chang PC. The potential of salivary biomarkers for predicting the sensitivity and monitoring the response to nonsurgical periodontal therapy: a preliminary assessment. J Periodontal Res. (2018) 53:545–54. 10.1111/jre.12544 29882262

[123] Wang L Lu W Ju W Yao W Shi C Yang X. The salivary metabolomics analyses reveal the Variable metabolites in distinct staging of periodontitis. BMC Oral Health. (2025) 25:480. 10.1186/S12903-025-05792-Y 40181365 PMC11966846

[124] Polz-Dacewicz M Strycharz-Dudziak M Dworzański J Stec A Kocot J. Salivary and serum IL-10, TNF-α, TGF-*β*, VEGF levels in oropharyngeal squamous cell carcinoma and correlation with HPV and EBV infections. Infect Agent Cancer. (2016) 11:45. 10.1186/s13027-016-0093-6 27547238 PMC4992298

[125] Korostoff A Reder L Masood R Sinha UK. The role of salivary cytokine biomarkers in tongue cancer invasion and mortality. Oral Oncol. (2011) 47:282–7. 10.1016/j.oraloncology.2011.02.006 21397550

[126] Al Shaar A Hamadeh O Ali A. Saliva and serum biomarkers in oral diseases: a case-control study. Medicine (Baltimore). (2024) 103:e41072. 10.1097/MD.0000000000041072 39969370 PMC11688012

[127] Surdu A Foia LG Luchian I Trifan D Tatarciuc MS Scutariu MM. Saliva as a diagnostic tool for systemic diseases—a narrative review. Medicina (B Aires). (2025) 61:243. 10.3390/medicina61020243 PMC11857487 40005360

[128] Kumar P Gupta S Das BC. Saliva as a potential non-invasive liquid biopsy for early and easy diagnosis/prognosis of head and neck cancer. Transl Oncol. (2024) 40:101827. 10.1016/j.tranon.2023.101827 38042138 PMC10701368

[129] Johnston W Rosier BT Artacho A Paterson M Piela K Delaney C. Mechanical biofilm disruption causes microbial and immunological shifts in periodontitis patients. Sci Rep. (2021) 11:9796. 10.1038/S41598-021-89002-Z 33963212 PMC8105330

[130] Li Y He X Luo G Zhao J Bai G Xu D. Innovative strategies targeting oral microbial dysbiosis: unraveling mechanisms and advancing therapies for periodontitis. Front Cell Infect Microbiol. (2025) 15:1556688. 10.3389/fcimb.2025.1556688 40370404 PMC12075390

[131] Messina BM Polizzi A Panuzzo C Belmonte A Angjelova A Fuochi V. Impact of periodontal host-modulation therapies on oral–gut microbiome axis in periodontitis patients with hematological diseases: a narrative review. Life. (2025) 15:1862. 10.3390/life15121862 41465800 PMC12734958

[132] Albagieh H Alshehri AZ Alduraywishi AS Aldaws A AlBalawi SS Abu Shaqqaf HF. Evaluation of salivary diagnostics: applications, benefits, challenges, and future prospects in dental and systemic disease detection. Cureus. (2025) 17:e77520. 10.7759/cureus.77520 39958008 PMC11830415

[133] Germain A Glass KA Eckert MA Giloteaux L Hanson MR. Temporal dynamics of the plasma proteomic landscape reveals maladaptation in ME/CFS following exertion. Mol Cell Proteomics. (2025) 24:101467. 10.1016/j.mcpro.2025.101467 41237904 PMC12757482

[134] Song J Wang C Zhao T Zhang Y Xing J Zhao X. Multi-omics approaches for biomarker discovery and precision diagnosis of prediabetes. Front Endocrinol (Lausanne). (2025) 16:1520436. 10.3389/fendo.2025.1520436 40162315 PMC11949806

[135] Ștefârță A Brătoiu MR Rădoi MA Mercuț V Ionescu M Scrieciu M. Assessment of salivary parameters—pH, buffering capacity and flow-associated with caries susceptibility. Diagnostics. (2026) 16:625. 10.3390/DIAGNOSTICS16040625 41750773 PMC12939177

[136] Akter R Asgor Moral MA Md K A. K. M. B. Biomimetic effect of saliva on human tooth enamel: a scanning electron microscopic study. Int J Dent. (2025) 2025:1664620. 10.1155/IJOD/1664620 39801838 PMC11724731

